# *﻿Cavariella* Del Guercio (Hemiptera, Aphidinae, Macrosiphini) in China, with a new species, new synonymies, and first country records

**DOI:** 10.3897/zookeys.1169.98552

**Published:** 2023-07-18

**Authors:** Ying Xu, Jing Chen, Li-Yun Jiang, Ge-Xia Qiao

**Affiliations:** 1 Key Laboratory of Zoological Systematics and Evolution, Institute of Zoology, Chinese Academy of Sciences, No. 1–5 Beichen West Road, Chaoyang District, Beijing 100101, China Institute of Zoology, Chinese Academy of Sciences Beijing China; 2 College of Life Science, University of Chinese Academy of Sciences, No. 19, Yuquan Road, Shijingshan District, Beijing 100049, China University of Chinese Academy of Sciences Beijing China

**Keywords:** Apiaceae, DNA barcode, key, morphology, new record, new synonym, *Salix*

## Abstract

The genus *Cavariella* is distinguished from other Macrosiphini genera (Aphididae, Aphidinae) because it has a supra-caudal process on abdominal tergite VIII which possesses two setae distally. It is Holarctic in distribution, and half of its species are Asian. The Chinese fauna of this genus, 17 species, have been restudied, morphologically and through DNA barcodes. As a result: *Cavariellahidaensis* Takahashi is transferred to *Elatobium*; *Cavariellasculptura* Qiao & Xu, **sp. nov.** is described from specimens collected on *Torilis* and *Cryptotaenia* (Apiaceae); *Cavariellacessana* Zhang, Chen, Zhong & Li, **syn. nov.** and *Cavariellalargispiracula* Zhang, Chen, Zhong & Li, **syn. nov.** are respectively junior synonyms of *Cavariellaaquatica* (Gillette & Bragg) and *Cavariellasapporoensis* Takahashi; *Cavariellagilgiana* Zhang, Chen, Zhong & Li and *Cavariellalhasana* Zhang are confirmed as valid species and complete descriptions are provided; *Cavariellabhutanensis* Chakrabarti & Das, *Cavariellanigra* Basu, *Cavariellapastinacae* (Linnaeus), and *Cavariellapustula* Essig are recorded for the first time from China. Additionally, keys for species of *Cavariella* known in China are provided and modifications to the key by Blackman and Eastop of aphid species on *Angelica* (Aphids on World’s Plants) are presented.

## ﻿Introduction

*Cavariella* is a genus of Macrosiphini (Hemiptera, Aphidinae) with three subgenera, *Cavaraiellia* Heinze, 1960, *Cavariella* Del Guercio, 1911, and *Cavariellinepicauda* Ivanovskaja, 1980. The genus is characterized by the presence of a supra-caudal process on abdominal tergite VIII which possesses two setae distally (Blackman and Eastop 2022). Most species of the genus alternate hosts between *Salix* and Apiaceae (Blackman and Eastop 2022). Species of the genus are distributed in the Holarctic and half of them can be found in Asia. In China, Tao (1964) first recorded the first four species of the genus, namely *Cavariellaaegopodii* (Scopoli), *C.araliae* Takahashi, *C.japonica* (Essig & Kuwana). and *C.salicicola* (Matsumura). Subsequently, Zhang and Zhong (1981) recorded *C.aegopodii* (Scopoli) and *C.lhasana* Zhang in Tibet, China. *Cavariellaangelicae* (Matsumura) and *C.sapporoensis* Takahashi were recorded in Hebei, China (Zhang and Zhong 1990). Zhang et al. (1999) recorded nine species in the genus around the northwest region of China, including *C.aegopodii* (Scopoli), *Cavariellaaquatica* (Gillette & Bragg), *Cavariellacessana* Zhang, Chen, Zhong & Li, *Cavariellagilgiana* Zhang, Chen, Zhong & Li, *C.konoi* Takahashi, *C.largispiracula* Zhang, Chen, Zhong & Li, *C.salicicola* (Matsumura), *C.yuzhongensis* Zhang, Chen, Zhong & Li and *C.zhangi* Zhang, Chen, Zhong & Li. Then, *C.yuzhongensis* Zhang, Chen, Zhong & Li and *C.zhangi* Zhang, Chen, Zhong & Li were respectively regarded as synonyms of *C.largispiracula* Zhang, Chen, Zhong & Li and *C.aquatica* (Gillette & Bragg) by Eastop and Blackman (2005). Qiao et al. (2005) described another species *C.cicutisucta* Qiao in Shanxi, China. Jiang et al. (2011) recorded five species in the genus around the northeast of China, namely *C.aegopodii* (Scopoli), *C.araliae* Takahashi, *C.konoi* Takahashi, *C.nipponica* Takahashi, and *C.salicicola* (Matsumura).

The diversity of the genus seems to be very high in China, but still lacks taxonomic research. Based on examination of specimens of *Cavariella* in China, the genus is systematically revised in this work; the genus includes 42 species in the world, of which 17 species can be found in China.

## ﻿Materials and methods

Aphid terminology in this paper generally follows Chakrabarti and Das (2009), Favret (2022), and Blackman and Eastop (2022). The order to record each specimen is as follows: the number of specimens, locality, collection date, collection number, host plant, collector.

In this paper, the following abbreviations are used:

**Ant. I, II, III, IV, V, VI** antennal segments I, II, III, IV, V, VI;

**Ant. Vb** or **VIb** base of antennal segment V or VI, respectively;

**PT** processus terminalis ×;

**Ant. IIIBD** basal diameter of antennal segment III;

**URS** ultimate rostral segment;

**BW URS** basal width of ultimate rostral segment;

**MW** hind tibia: mid-width of hind tibia;

**HT II** second hind tarsal segment;

**SIPH** siphunculus;

**BW SIPH** basal width of siphunculus;

**DW SIPH** distal width of siphunculus;

**MW SIPH** mid-width of siphunculus;

**BW Cauda** basal width of cauda;

**ABD TERG VIII** abdominal tergite VIII;

**ap. viv. fem.** apterous viviparous female;

**ala. viv. fem.** alate viviparous female.

DNA barcodes of COI were obtained from the Chinese species. COI DNA barcodes were obtained from specimens belonging to samples listed in Table 1. The methods of extracting DNA and PCR thermal regime followed those of Chen et al. (2015). Sequences were assembled by SeqMan II (DNAStar, Inc., Madison, WI, USA) with inspection and manual editing, and then were examined using BLAST to confirm the sequences were highly similar to other aphid sequences. All sequences were deposited in GenBank (Table 1). Multiple alignments were performed with ClustalW (Thompson et al. 1994) and then verified manually. Pairwise genetic distances and Neighbor-joining (NJ) tree for the COІ gene were estimated using MEGX (Kumar et al. 2018) under Kimura’s two-parameter (K2P) model (Kimura 1980). Bootstrap analyses were performed with 1000 replicates.

**Table 1.** Voucher and GenBank accession numbers for aphid samples used in the molecular analyses.

Species	Voucher number	Collection locality	Host plant	COI
*Cavariellaaegopodii*	BIOUG27096-B11	Canada	/	MF835290.1*
	BIOUG10644-D06	Canada	/	KR344313.1*
	CNC#HEM068401	Canada	/	HQ971309.1*
	CNC#HEM063948	Canada	/	GU668356.1*
	CNC#HEM063245	Canada	/	GU667510.1*
	BIOUG20138-H04	Canada	/	MF838462.1*
	CNC#HEM063243	Canada	/	GU667509.1*
	40940	CHINA: Tibet	*Salix*	OP956128
	50266	CHINA: Yunnan	*Salix*	OP956143
	51884	CHINA: Tibet	*Salix*	OP956151
*Cavariellaangelicae*	50575	CHINA: Sichuan	*Salix*	OP956145
	50588	CHINA: Sichuan	*Salix*	OP956146
	51435	CHINA: Sichuan	*Salix*	OP956148
*Cavariellaaquatica*	32742	CHINA: Tibet	Hordeumvulgarevar.coeleste	OP956121
	32744	CHINA: Tibet	*Salix*	OP956122
*Cavariellaaraliae*	HL81	CHINA	*Aralia*	MH821599.1*
	HLshujia451	CHINA	*Aralia*	MH821600.1*
	HLshujia452	CHINA	*Aralia*	MH821601.1*
	HLshujia93	CHINA	*Aralia*	MH821602.1*
	45335	CHINA: Hubei	*Aralia*	OP956137
*Cavariellabhutanensis*	32711	CHINA: Tibet	*Salix*	OP956120
	51895	CHINA: Tibet	*Salix*	OP956152
*Cavariellacicutisucta*	33648	CHINA: Guizhou	Apiaceae	OP956123
*Cavarielladigitata*	BIOUG07811-D11	Canada	/	KR563910.1*
	CNC#HEM070376	Canada	/	JF883634.1*
*Cavariellahidaensis*	22552	CHINA: Sichuan	unknown	OP956114
	50569	CHINA: Sichuan	*Salix*	OP956144
	51438	CHINA: Sichuan	*Salix*	OP956149
*Cavariellagilgiana*	51429	CHINA: Sichuan	*Salix*	OP956147
*Cavariellajaponica*	31362	CHINA: Sichuan	Apiaceae	OP956119
	36809	CHINA: Hubei	Apiaceae	OP956124
	43839	CHINA: Sichuan	Apiaceae	OP956135
	44015	CHINA: Sichuan	Apiaceae	OP956136
*Cavariellakonoi*	CNC#HEM064274	Canada	/	HM416733.1*
	CNC#HEM064234	Canada	/	HM416692.1*
	CNC#HEM007238	Canada	/	HQ970747.1*
	CNC#HEM070643	Canada	/	JF883690.1*
	CNC#HEM070349	Canada	/	JF883617.1*
	CNC#HEM070380	Canada	/	JF883638.1*
	CNC#HEM070364	Canada	/	JF883626.1*
	CNC#HEM070249	Canada	/	JF883546.1*
	41101	CHINA: Jilin	*Salix*	OP956129
	41195	CHINA: Jilin	*Salix*	OP956132
*Cavariellanigra*	36849	CHINA: Hubei	Apiaceae	OP956126
	41140	CHINA: Jilin	Apiaceae	OP956130
	43081	CHINA: Shaanxi	Apiaceae	OP956134
*Cavariellanipponica*	30077	CHINA: Sichuan	unknown	OP956116
	31000	CHINA: Beijing	*Salix*	OP956118
	51630	CHINA: Sichuan	*Salix*	OP956150
*Cavariellapastinacae*	CNC#HEM007501	Canada	/	HQ970726.1*
	CNC#HEM063678	Canada	/	GU668687.1*
	CNC#HEM064086	Canada	/	GU668257.1*
	CNC#HEM063641	Canada	/	GU668529.1*
	55639	CHINA: Xinjiang	Apiaceae	OP956153
*Cavariellapustula*	50011	CHINA: Beijing	*Salix*	OP956142
*Cavariellasalicicola*	HLshujia506	CHINA	*Salix*	MH821603.1*
	HLshujia607	CHINA	*Salix*	MH821604.1*
	30855	CHINA: Beijing	*Salix*	OP956117
*Cavariellasapporoensis*	/	Korea	/	GU978782.1*
	/	Korea	/	KX631497.1*
	41184	CHINA: Jilin	Apiaceae	OP956131
	42064	CHINA: Beijing	Apiaceae	OP956133
	45505	CHINA: Beijing	Apiaceae	OP956139
	45517	CHINA: Beijing	Apiaceae	OP956140
	45522	CHINA: Beijing	Apiaceae	OP956141
*Cavariellasculptura* sp. nov.	36840	CHINA: Hubei	Apiaceae	OP956125
	36853	CHINA: Hubei	Apiaceae	OP956127
	45394	CHINA: Hubei	Apiaceae	OP956138
*Cavariellatheobaldi*	CNC#HEM003818	Canada	/	KR032549.1*
	/	/	/	KF639272.1*
	CNC#HEM063229	Canada	/	GU667508.1*
	CNC#HEM007231	Canada	/	EU701562.1*
	CNC#HEM010567	Canada	/	EU701563.1*
*Elatobiumabietinum*	/	/	/	MW441485.1*
	CNC#HEM063197	Canada	/	GU667432.1*
	BIOUG09976-G02	Canada	/	KR343676.1*
*Elatobiumsalicifoliae*	25750	CHINA	*Salix*	KC286721.1*

*Sequences downloaded from GenBank.

### ﻿Specimen depositories

The holotype and some paratypes of the new species and other specimens examined are deposited in the National Animal Collection Resource Center, Institute of Zoology, Chinese Academy of Sciences, Beijing, China. The other paratypes of the new species are deposited in the Natural History Museum, London, UK (**NHMUK**, indicated in the text).

## ﻿Taxonomic account

### ﻿ Cavariella

Taxon classificationAnimaliaHemipteraAphididae

Del Guercio, 1911

E5BDA521-D9F3-5962-98B9-FE75827E9768

Cavariella Del Guercio, 1911: 323. Type species: Aphispastinacae Linnaeus, 1758.

#### Diagnosis.

Body dorsum with various ornaments: wrinkles, irregularly circular or semicircular sculptures, small papillate tubercles. Frons convex. Antennae shorter than body length, without secondary rhinaria in apterae; in alatae, Ant. III with circular secondary rhinaria distributed over the whole segment, sometimes the secondary rhinaria protruded, Ant. IV and V usually with circular secondary rhinaria. ABD TERG VIII with a spinal supra-caudal process varying from a very long conical process to an indistinct swelling and possessing two setae distally; in alatae, the supra-caudal process is reduced and wart-like. Siphunculus clavate, swollen distally, sometimes elongated cylinder and not swollen, with imbrications. Cauda elongate conical, conical, or tongue-shaped.

#### Taxonomy.

The genus *Cavariella* has been divided into three subgenera, *Cavaraiellia* Heinze, Cavariellinepicauda Ivanovskaja, and the nominate subgenus Cavariella Del Guercio.

#### Biology.

Most species of the genus characteristically alternate hosts, *Salix* species being the primary host, and plant species of diverse families, frequently Apiaceae the secondary host. Aphids colonize the aerial parts of plants, mainly tender ones.

### ﻿Key to the subgenera of *Cavariella*

**Table:** 

1	Siphunculus clavate, obliquely truncated at tip, without flange; cauda long conical, with 7 or 8 setae	***Cavaraiellia***
–	Siphunculus clavate or cylindrical, with distinct flange; cauda conical or tongue-shaped, with 4–6 or > 8 setae	**2**
2	ABD TERG VIII with distinct supra-caudal process, as long as or longer than Ant. II; cauda conical with 4–6 setae	***Cavariella***
–	ABD TERG VIII with indistinct supra-caudal process, shorter than Ant. II; cauda tongue-shaped with > 8 setae	***Cavariellinepicauda***

### ﻿Subgenus Cavaraiellia

Taxon classificationAnimaliaHemipteraAphididae

Heinze, 1960

E704CE26-4681-5EAE-B49C-5B0BA10135F1

CavaraielliaHeinze 1960: 810. Type species: Cavariellahillerislambersi Ossiannilsson (= aquatica).

#### Diagnosis.

Frons convex. ABD TERG VIII with short conical supra-caudal process; siphunculus clavate, obliquely truncated at tip, without flange, the pore short than the distal width; cauda long conical, with seven or eight setae.

#### Comment.

The subgenus only contains one species. *Cavariellacessana* Zhang, Chen, Zhong & Li, 1999 is considered as a junior synonym of *Cavariellaaquatica* (Gillette & Bragg, 1916).

### ﻿ Cavariella (Cavaraiellia) aquatica

Taxon classificationAnimaliaHemipteraAphididae

(Gillette & Bragg, 1916)

C336941E-9C62-55E3-A0DA-425B65F7A692

Figs 1 , 2 , 32A–C

Siphocoryne aquatica Gillette & Bragg, 1916: 447.

#### Types examined.

***Holotype*** and ***paratypes*** of *Cavariellacessana* Zhang, Chen, Zhong & Li, 1999: one ap. viv. fem. and one ala. viv. fem., **China: Xinjiang** (Korla City), 19.VI.1989, No. 9391, on *Salix*, coll. G.X. Zhang and T.S. Zhong (Zhang et al 1999).

#### Other specimens examined.

One ap. viv. fem., **Tibet** (Xigaze City), 17.VII.2022, No. 51892-1-1, on *Salix*, coll. Y. Xu; one ap. viv. fem.; **Tibet** (Xigaze City), 26.VII.2014, No. 32740-1-1, on *Salix*, coll. J. Chen and X.C. Zhu; one ap. viv. fem., **Tibet**, 23.VI.2016, No. 37396-1-1, on *Salix*, coll. F.F. Niu; one ap. viv. fem. and one ala. viv. fem., **Tibet** (Linzhi City), 23.VI.2014, No. 37394-1-1, on *Salix*, coll. F.F. Niu; one ap. viv. fem. and one ala. viv. fem. (slides), one ap. viv. fem. (COI: OP956122), **Tibet** (Xigaze City), 26.VII.2014, No. 32744-1-1, on *Salix*, coll. J. Chen and X.C. Zhu; one ap. viv. fem., **Tibet** (Xigaze City), 16.VII.2014, No. 32664-1-1, on Hordeumvulgarevar.coeleste, coll. J. Chen, X.C. Zhu, X.H. Lou; two ap. viv. fems. (slides) and one ap. viv. fem. (COI: OP956121), **Tibet** (Xigaze City), 26.VII.2014, No. 32742-1-1, on Hordeumvulgarevar.coeleste, coll. J. Chen, X.C. Zhu, X.H. Lou; one ap. viv. fem. and one ala. viv. fem., **Tibet** (Linzhi City), 23.VI.2016, No. 37393-1-1, on Poaceae, coll. F.F. Niu; nine ap. viv. fems., **Xinjiang**, 11.VII.1977, No. 6892, on *Salix*, coll. Y.H. Han (Zhang and Zhong 1985); two ap. viv. fems., **Xinjiang**, 11.VII.1977, No. 6644, on *Salix*, coll. Y.H. Han (Zhang and Zhong 1985); two ap. viv. fems., **Qinghai**, 14.VI.1997, No. 11430, on *Salix*, coll. X.L. Chen (Zhang et al. 1999); eighteen ap. viv. fems., **Qinghai**, 13.VI.1997, No. 11417, on *Salix*, coll. X.L. Chen (Zhang et al. 1999).

#### Diagnosis.

**Figure 1.**
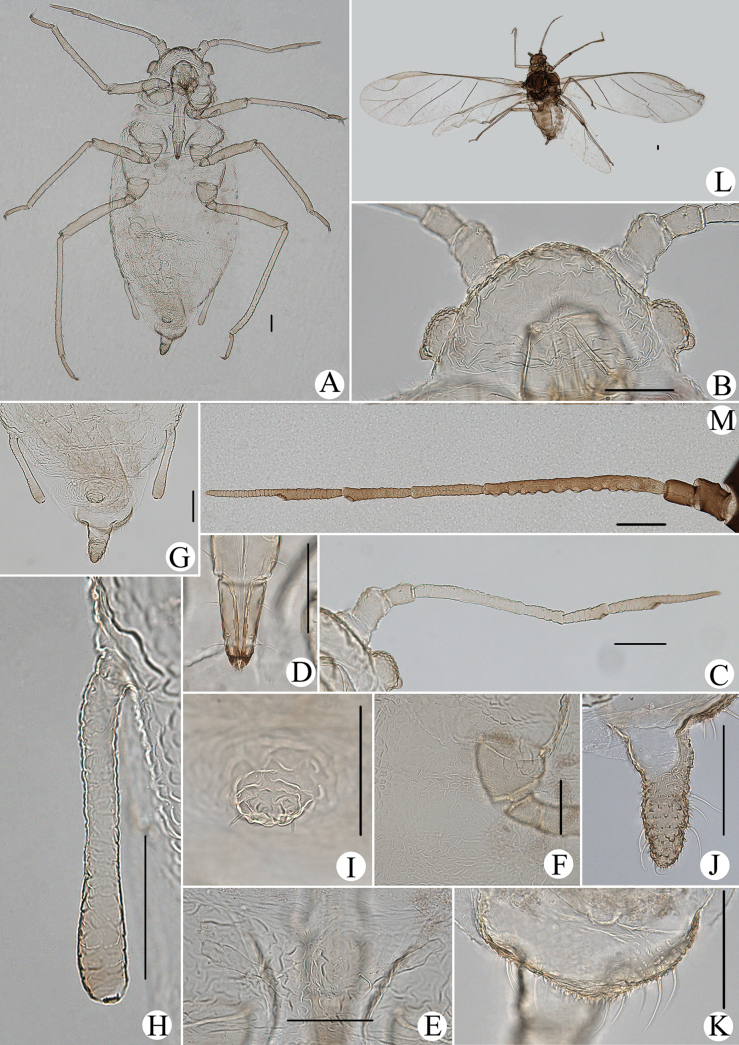
Cavariella (Cavaraiellia) aquatica (Gillette & Bragg). Apterous viviparous female: **A** dorsal view of body **B** dorsal view of head **C** antenna **D** ultimate rostral segment **E** mesosternal furca **F** sculptures of abdominal tergites **G** abdominal tergites VI–VII **H** siphunculus **I** supra-caudal process on abdominal tergite VIII **J** cauda **K** anal plate. Alate viviparous female: **L** dorsal view of body **M** antenna. Scale bars: 0.10 mm.

Body dorsum is covered with wavy or irregular circular wrinkles; ABD TERG VIII with short conical supra-caudal process, blunt at apex (Fig. 1G, I); siphunculus clavate, obliquely truncated at tip, without flange (Fig. 1H) (Gillette and Bragg 1916; Zhang and Zhong 1985; Zhang et al. 1999).

#### Comments.

**Figure 2.**
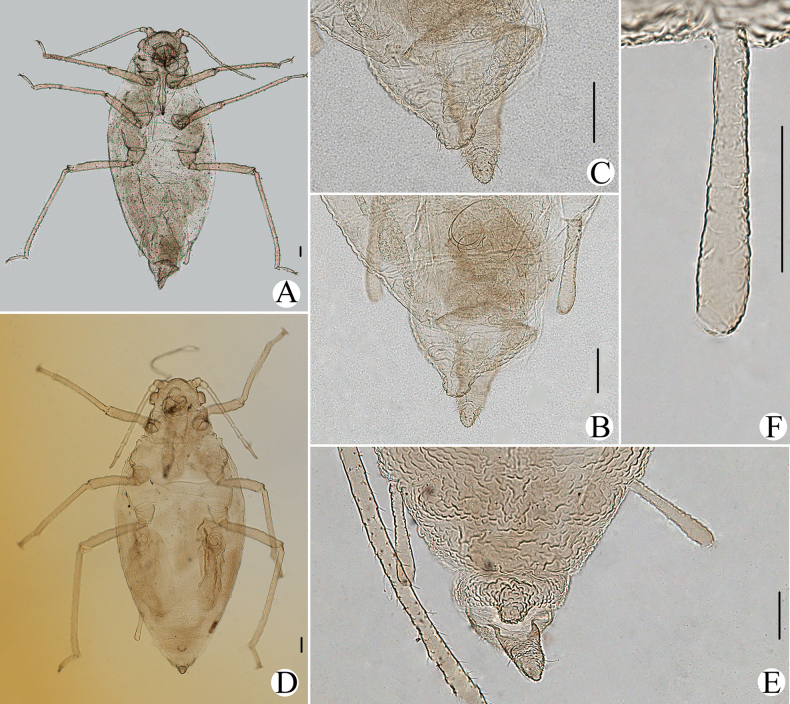
Cavariella (Cavaraiellia) aquatica (Gillette & Bragg) feeding on Poaceae: **A** dorsal view of body **B** abdominal tergites VI–VIII **C** supra-caudal process on abdominal tergite VIII. *Cavariellacessana* Zhang, Chen, Zhong & Li, 1999, syn. nov. **D** dorsal view of body **E** abdominal tergites VI–VIII **F** siphunculus. Scale bars: 0.10 mm.

The species migrates between *Salix* and Poaceae, Cyperaceae, or Juncaceae, and the supra-caudal process has some variations between the host plants. The population feeding on *Salix* has a broad, short, conical supra-caudal process (Fig. 1I), while those on secondary host plants have a slightly larger supra-caudal process; sometimes the ABD TERG VIII bends backward to a triangular supra-caudal process (Fig. 2B, C), so there can be some misidentifications to this species. Zhang et al. (1999) described *Cavariellacessana* (Fig. 2D–F) based on apterae and alatae feeding on *Salix*. After checking many specimens of the two species in China, they were found to be the same species feeding on different host plants. Additionally, the molecular data supported that *C.aquatica* and *C.cessana* are the same species (Fig. 38). Therefore, *Cavariellacessana* Zhang, Chen, Zhong & Li, 1999 should be considered as a junior synonym of *Cavariellaaquatica* (Gillette & Bragg, 1916).

#### Biology.

Primary host plant: *Salix*; secondary host plant: Poaceae, Cyperaceae, or Juncaceae. The species feeds on upper sides of leaves and tender tips of the primary host plant (Fig. 32A, B). As to the secondary host plant, the species feeds on a water-grass *Catabrosaaquatica* (L.) growing on seeped land and usually was found in the water along the margins of ditches in USA (Gillette and Bragg 1916). In China, the species feeds on the upper surfaces of Hordeumvulgarevar.coeleste L. (Fig. 32C).

#### Distribution.

China (Qinghai, Tibet, Xinjiang), Afghanistan, Canada, Europe, India, Kazakhstan, Pakistan, USA.

### ﻿Subgenus Cavariella

Taxon classificationAnimaliaHemipteraAphididae

Del Guercio, 1911

7D26D523-4E86-548A-A5B9-67EAB4A84730

Cavariella Del Guercio, 1911: 323. Type species: Aphispastinacae Linnaeus.

#### Diagnosis.

ABD TERG VIII with distinct supra-caudal process, elongate conical, conical, or triangular, as long as or longer than Ant. II. Siphunculus mostly clavate, swollen distally, sometimes long cylindrical not swollen, with distinct flange and imbrications. Cauda conical mostly with 4–6 setae, sometimes with 5–9 setae.

#### Comment.

The nominate subgenus contains thirty-six species, including one new species. There are fourteen species in China, and *Cavariellabhutanensis* Chakrabarti & Das, 2009, *C.nigra* Basu, 1964, *C.pastinacae* (Linnaeus, 1758), and *C.pustula* Essig, 1937 are recorded in China for the first time. Two endemic species in China, *Cavariellagilgiana* Zhang, Chen, Zhong & Li, 1999 and *Cavariellalhasana* Zhang, 1981, are re-described in this work.

### ﻿ Cavariella aegopodii

Taxon classificationAnimaliaHemipteraAphididae

(Scopoli, 1763)

F51C3CDB-5122-5CC9-9E66-B675C1C9D56D

Figs 3 , 4 , 32D

Aphis aegopodii Scopoli, 1763: 137.

#### Specimens examined.

Six ap. viv. fems. and four ala. viv. fems., **China: Yunnan**, 25.V.1980, No. 7157, on *Foeniculumvulgare*, coll. T.S. Zhong and L.Y. Wang (Zhang and Zhong 1984); one ap. viv. fem., **Gansu** (Zhangxian County), 24.VII.1986, No. 8501-3-4, on *Salix*, coll. G.X. Zhang, T.S. Zhong and J.H. Li (Zhang et al. 1999); one ap. viv. fem. (slide) and one ap. viv. fem. (COI: OP956128), **Tibet** (Lasa City), 23.VIII.2017, No. 40940-1-1, on *Salix*, coll. X.Y. Luo; two ap. viv. fems. (slides) and one apterous female (COI: OP956143), **Yunnan** (Lijiang City), 26.V.2021, No. 50266-1-1, on *Salix*, coll. T.Y. Liu and S. Xu; two ap. viv. fems. (slides) and one apterous female (COI: OP956151), **Tibet** (Xigaze City), 16.VII.2021, No. 51884-1-1, on *Salix*, coll. Y. Xu; three ala. viv. fems. and one ap. viv. fem., **Qinghai** (Xining City), 5.VI.1997, No. 11333, on *Salix*, coll. X.L. Chen; one ala. viv. fem. and one ap. viv. fem., **Sichuan**, 24.VIII.2013, No. 30092-1-1, on unknown host plant, coll. R. Chen.

#### Diagnosis.

**Figure 3.**
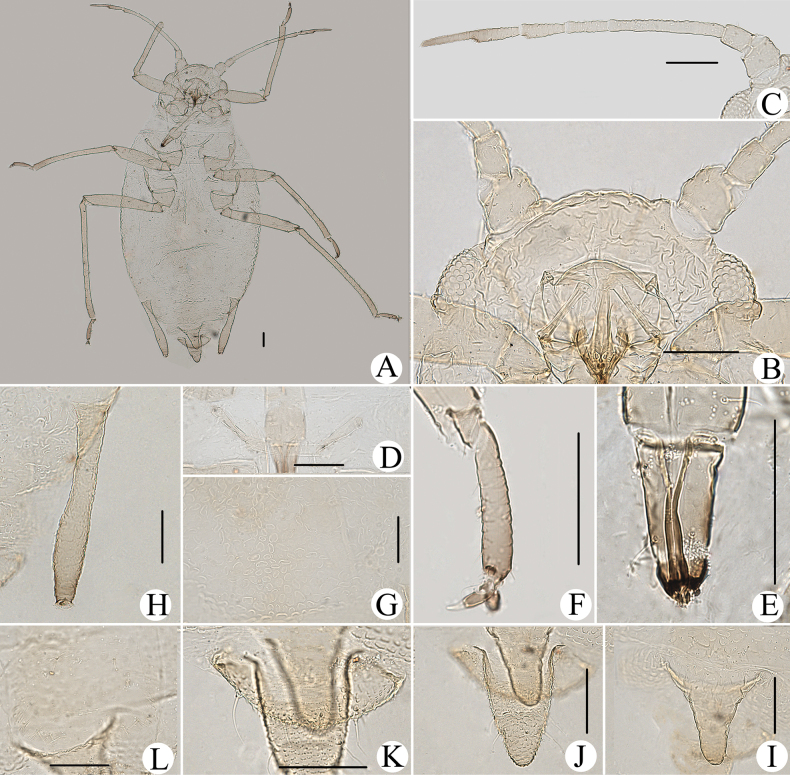
*Cavariellaaegopodii* (Scopoli). Apterous viviparous female: **A** dorsal view of body **B** dorsal view of head **C** antenna **D** mesosternal furca **E** ultimate rostral segment **F** second hind tarsal segment **G** sculptures of abdominal tergites **H** siphunculus **I** supra-caudal process on abdominal tergite VIII **J** cauda **K** anal plate **L** genital plate. Scale bars: 0.10 mm.

URS shorter than HT II and without accessory setae (Fig. 3E, F); PT 0.85–1.14× Ant. VIb, mostly longer than Ant. VIb (Fig. 3C); supra-caudal process longer than 1/2 of cauda but rarely extending beyond tip of cauda (Fig. 3I) (Zhang and Zhong 1984; Zhang et al. 1999).

**Figure 4.**
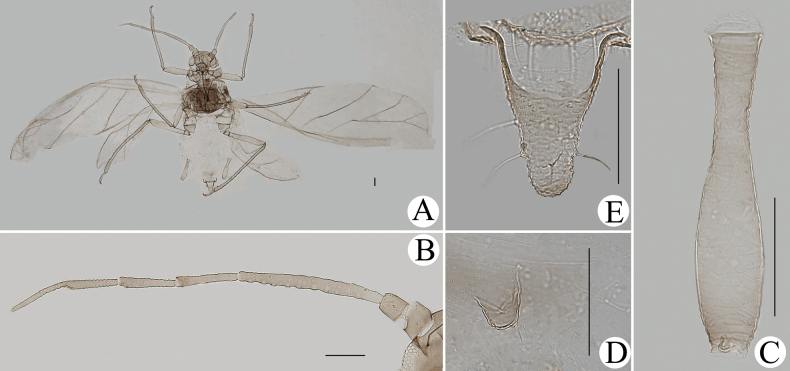
*Cavariellaaegopodii* (Scopoli). Alate viviparous female: **A** dorsal view of body **B** antenna **C** siphunculus **D** supra-caudal process on abdominal tergite VIII **E** cauda. Scale bars: 0.10 mm.

#### Biology.

Primary host plant: *Salix*; secondary host plant: Apiaceae. The species feeds on leaves and tender tips of *Salix* (Fig. 32D). The species is a major pest of cultivated Apiaceae, and it feeds on leaves and umbels of many genera in Apiaceae (Blackman and Eastop 2022).

#### Distribution.

China (Heilongjiang, Gansu, Qinghai, Sichuan, Taiwan, Tibet, Xinjiang, Yunnan, Zhejiang) (Zhang et al 1992; Jiang et al 2011), Canada, Europe, USA.

### ﻿ Cavariella angelicae

Taxon classificationAnimaliaHemipteraAphididae

(Matsumura, 1918)

28C97E3D-3273-5F25-B642-FE1C045B3326

Figs 5 , 32E

Metaphis angelicae Matsumura, 1918: 1.

#### Specimens examined.

Two ap. viv. fems. (slides) and one ap. viv. fem. (COI: OP956148), **China: Sichuan** (Ganzi City), 15.VI.2021, No. 51435-1-1, on *Salix*, coll. T.Y. Liu and S. Xu; one ap. viv. fem. and one ala. viv. fem. (slides), one ap. viv. fem. (COI: OP956145), **Sichuan** (Ganzi City), 12.VI.2021, No. 50575-1-1, on *Salix*, coll. T.Y. Liu and S. Xu; two ap. viv. fems. and two ala. viv. fems. (slides), one ap. viv. fem. (COI: OP956146), **Sichuan** (Ganzi City), 12.VI.2021, No. 50588-1-1, on *Salix*, coll. T.Y. Liu and S. Xu; one ap. viv. fem., **Hebei**, 6.V.2021, No. 49999-2-1, on *Salix*, coll. G.X. Qiao.

#### Diagnosis.

**Figure 5.**
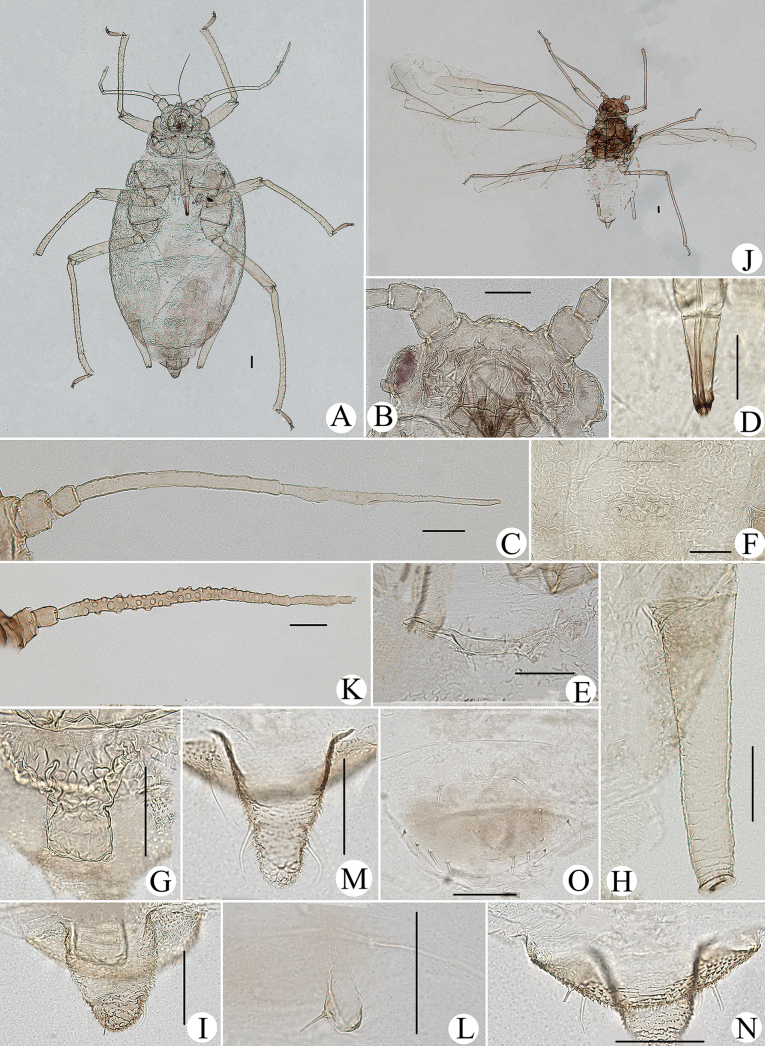
*Cavariellaangelicae* (Matsumura). Apterous viviparous female: **A** dorsal view of body **B** dorsal view of head **C** antenna **D** ultimate rostral segment **E** mesosternal furca **F** sculptures of abdominal tergites **G** supra-caudal process on abdominal tergite VIII **H** siphunculus **I** cauda. Alate viviparous female: **J** dorsal view of body **K** antennal segments I–IV **L** supra-caudal process on abdominal tergite VIII **M** cauda **N** anal plate **O** genital plate. Scale bars: 0.10 mm.

Antennae 5-segmented (Fig. 5C), PT 1.95–2.39× Ant. Vb; URS long wedge-shaped (Fig. 5D), 1.15–1.36× HT II; ABD TERG VIII with short rectangular supra-caudal process (Fig. 5G); SIPH cylindrical not swollen (Fig. 5H); cauda short tongue-shaped (Fig. 5I), with four or five setae (Matsumura 1918; Miyazaki 1971; Zhang and Zhong 1990).

#### Comment.

The species resembles *Cavarielladigitata* and *Cavariellatheobaldi* in SIPH being long and cylindrical, and not swollen, supra-caudal process short. But the species differs from *C.digitata* as follows: URS long and wedge-shaped, distinctly longer than HT II, 1.15–1.36× HT II (*C.digitata*: URS wedge-shaped, 1.00–1.10× HT II); supra-caudal process rectangular, blunt with two setae distally (*C.digitata*: supra-caudal process triangular, with two setae distally and 1–3 short setae basally); PT 3.00× Ant. Vb (*C.digitata*: PT 1.25–1.45× Ant. Vb). The species differs from *C.theobaldi* as follows: antennae 5-segmented (*C.theobaldi*: antennae 6-segmented); URS long wedge-shaped, longer than cauda (*C.theobaldi*: URS wedge-shaped, shorter than cauda) (Miyazaki 1971).

#### Biology.

The species feeds on tender tips of Apiaceae (*Angelica*, *Heracleum*, *Pastinaca*, *Sium*) (Miyazaki 1971; Blackman and Eastop 2022). In China, the species feeds on *Salix* (Fig. 32E), *Heracleum*, and *Angelica*. Hence, the species may be heteroecious holocyclic; *Salix* is the primary host plant and Apiaceae is the secondary host plant.

#### Distribution.

China (Hebei, Sichuan), Japan, Kazakhstan, Korea, Russia.

### ﻿ Cavariella araliae

Taxon classificationAnimaliaHemipteraAphididae

Takahashi, 1921

5AECEA90-4560-5CF7-B1A3-48A63CD28E49

Figs 6 , 7 , 32F

Cavariella araliae Takahashi, 1921: 37.

#### Specimens examined.

Two ap. viv. fems., **China: Guizhou** (Xishui County), 30.V.2000, No. 12518-1-1, on *Aralia*, coll. G.X. Qiao; two ap. viv. fems., **Sichuan** (Chengdu City), 21.VIII.2018, No. 43979-1-1, on *Aralia*, coll. Y. Xu and J.F. Ji; one ap. viv. fem., **Guizhou** (Fanjing Mountain), 1.VIII.2014, No. 33707-1-1, on *Aralia*, coll. Y.Q. Li and F.F. Niu; five ap. viv. fems. and three ala. viv. fems., **Hainan** (Linshui County), 7.IV.2015, No. 32504-1-1, No. 32505-1-1, No. 32507-1-1, No. 32503-1-1, on *Aralia*, coll. R. Chen; two ap. viv. fems., **Yunnan** (Jinping County), 17.IV.2018, No. 42356-1-1, on *Aralia*, coll. Y. Xu; three ap. viv. fems., **Hunan** (Ningyuan County), 27.V.2017, No. 39559-1-1, No. 39557-1-1, on *Aralia*, coll. C.C. Du and K. Hao; one ap. viv. fem., **Guangdong** (Shaoguan City), 7. VI.2017, No. 39667-1-1, on *Aralia*, coll. C.C. Du and K. Hao; two ap. viv. fems. (slides) and one ap. viv. fem. (COI: OP956137), **Hubei** (Yien County), 30.IV.2019, No. 45335-1-1, on *Aralia*, coll. X. L. Zhang; one ap. viv. fems., **Sichuan** (Leshan City), 7.VI.2014, No. 31318-1-1, on *Salix*, coll. Y. Wang and X. J Tang.

#### Diagnosis.

**Figure 6.**
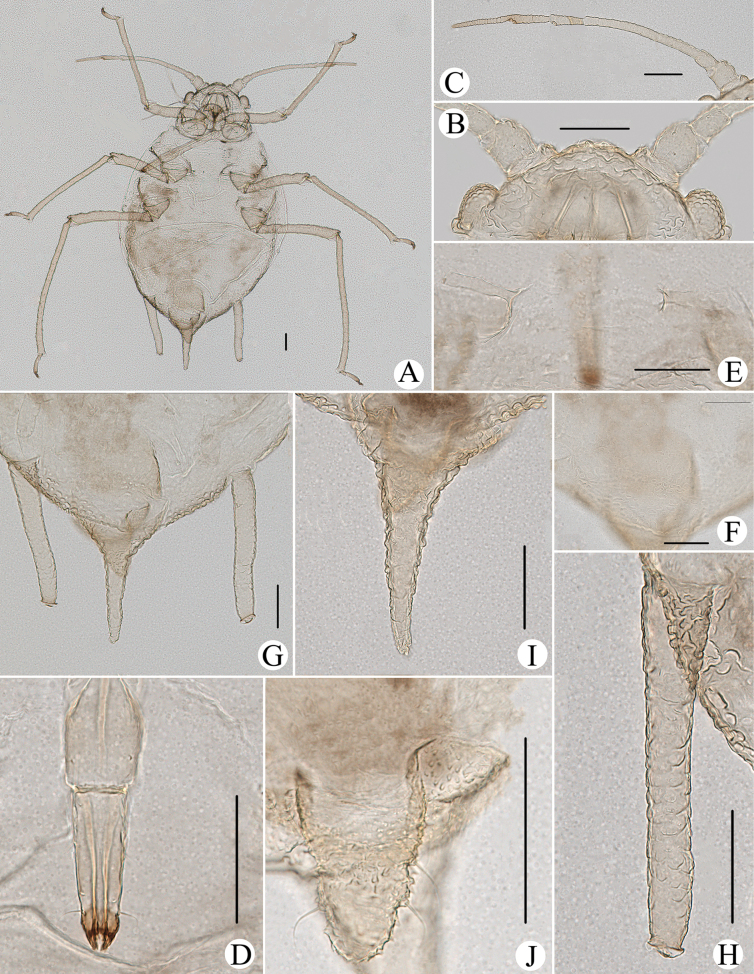
*Cavariellaaraliae* Takahashi. Apterous viviparous female: **A** dorsal view of body **B** dorsal view of head **C** antenna **D** ultimate rostral segment **E** mesosternal furca **F** sculptures of abdominal tergites **G** abdominal tergites VI–VIII **H** siphunculus **I** supra-caudal process on abdominal tergite VIII **J** cauda. Scale bars: 0.10 mm.

Supra-caudal long conical, pointed apex, much longer than cauda (Fig. 6G, I); antennae 5-segmented (Fig. 6C), PT 1.59× Ant. Vb; URS wedge-shaped (Fig. 6D), 1.40× HT II (Takahashi 1921; Jiang et al. 2011).

**Figure 7.**
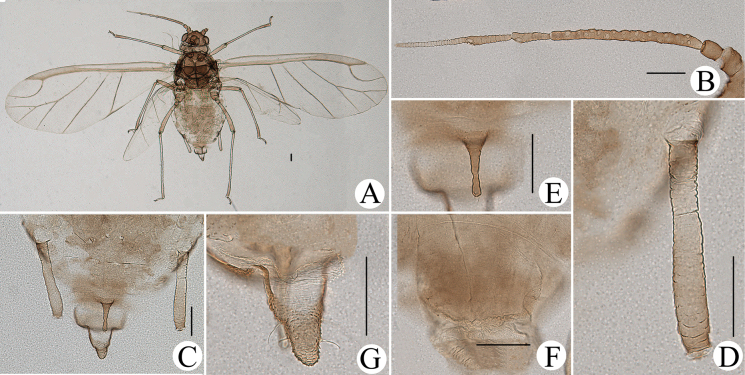
*Cavariellaaraliae* Takahashi. Alate viviparous female: **A** dorsal view of body **B** antenna **C** abdominal tergites VI–VIII **D** siphunculus **E** supra-caudal process on abdominal tergite VIII **F** genital plate **G** cauda. Scale bars: 0.10 mm.

#### Biology.

The species mostly feeds on stems and shoots of Araliaceae (Fig. 32F) (*Aralia*, *Schleffera*, *Tetrapanax*), and be found also feeding on *Salix* (Blackman and Eastop 2022). The species may migrate between *Salix* and Araliaceae.

#### Distribution.

China (Guangdong, Guizhou, Hainan, Henan, Hunan, Jiangsu, Jilin, Liaoning, Sichuan, Taiwan, Yunnan, Zhejiang) (Jiang et al. 2011), Japan, Korea, Russia.

### ﻿ Cavariella bhutanensis

Taxon classificationAnimaliaHemipteraAphididae

Chakrabarti & Das, 2009

45CE2AA1-D09A-50F9-95C7-B39013DC2F88

Figs 8 , 9 , 32G

Cavariella bhutanensis Chakrabarti & Das, 2009: 39.

#### Specimens examined.

One ala. viv. fem., **China: Tibet** (Nyalam County), 9.VIII.2010, No. 25818-1-1, on Apiaceae, coll. Y. Wang; one ala. viv. fem. and two ap. viv. fems., **Tibet** (Nyingchi City), 3.VIII.2010, No. 25756-1-1, on Apiaceae, coll. G.X. Qiao; one ap. viv. fem. (slide) and one ap. viv. fem. (COI: OP956120), **Tibet** (Nyalam County), 22.VII.2014, No. 32711-1-1, on *Salix*, coll. J. Chen and X.C. Zhu; one ap. viv. fem., **Tibet** (Shannan City), 9.VI.2016, No. 37289-1-1, on *Salix*, coll. F.F. Niu; one ap. viv. fem., **Tibet** (Jilong County), 24.VII.2014, No. 32732-1-1, on *Salix*, coll. J. Chen and X.C. Zhu; two ap. viv. fems. (slides) and one ap. viv. fem. (COI: OP956152), **Tibet** (Bailang County), 17.VII.2021, No. 51895-1-1, on *Salix*, coll. Y. Xu; two ap. viv. fems., **Tibet** (Pulan County), 31.VII.2021, No. 52077-1-1, on *Salix*, coll. Y. Xu; two ap. viv. fems., **Tibet** (Jilong County), 31.VII.2021, No. 52011-1-1, on Apiaceae, coll. Y. Xu.

#### Diagnosis.

**Figure 8.**
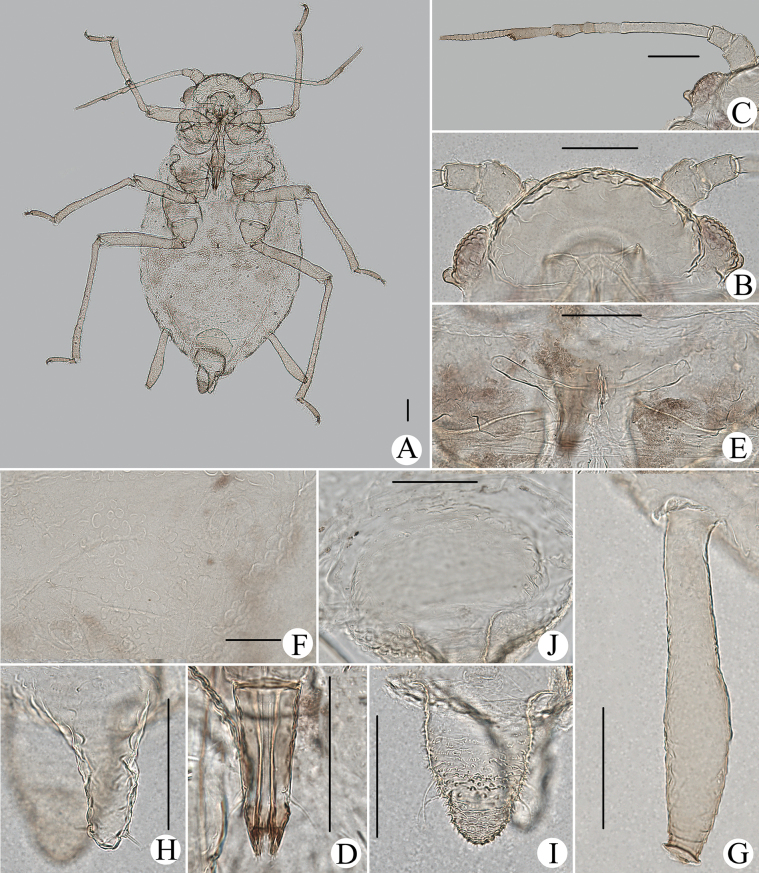
*Cavariellabhutanensis* Chakrabarti & Das. Apterous viviparous female: **A** dorsal view of body **B** dorsal view of head **C** antenna **D** ultimate rostral segment **E** mesosternal furca **F** sculptures of abdominal tergites **G** siphunculus **H** supra-caudal process on abdominal tergite VIII **I** cauda **J** genital plate. Scale bars: 0.10 mm.

PT 1.31–1.72× Ant. VIb; URS 0.11–0.15 mm, long wedge-shaped (Fig. 8D), 1.75–2.68× BW URS, 0.91–1.17× HT II; ABD TERG VIII with conical supra-caudal (Fig. 8H), 0.65–1.14× cauda; siphunculus 2.00–2.58× of cauda (Chakrabarti and Das 2009).

#### Comment.

The species is recorded in China for the first time, and with some variations from the original description as follows: PT 1.27–1.40× Ant. VIb; URS 1.15–1.28× HT II; supra-caudal process 1.05–1.14× cauda in Bhutan.

**Figure 9.**
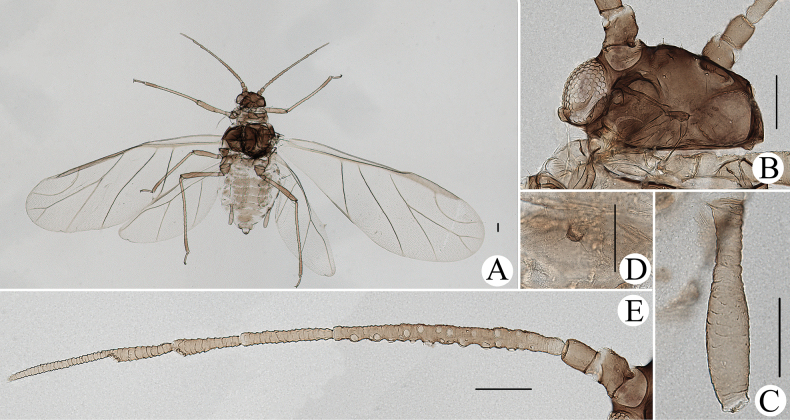
*Cavariellabhutanensis* Chakrabarti & Das. Alate viviparous female: **A** dorsal view of body **B** dorsal view of head **C** siphunculus **D** supra-caudal process on abdominal tergite VIII **E** antenna. Scale bars: 0.10 mm.

The species resembles *Cavariellaaegopodii* but differs as follows: PT 1.31–1.72× Ant. VIb (*C.aegopodii*: PT 1.12× Ant. VIb); URS elongate wedge-shaped, 0.91–1.17× HT II (*C.aegopodii*: URS wedge-shaped, shorter than HT II).

#### Biology.

The species was found feeding on *Salix* (Fig. 32G) and Apiaceae in China, and it also been described feeding on *Salix* in Bhutan (Chakrabarti and Das 2009). So, the species may migrate between the two plants.

#### Distribution.

China (Tibet), Bhutan.

### ﻿ Cavariella gilgiana

Taxon classificationAnimaliaHemipteraAphididae

Zhang, Chen, Zhong & Li, 1999

4651BE6F-2911-5156-9126-A5D1054BA3FB

**Figure 10.**
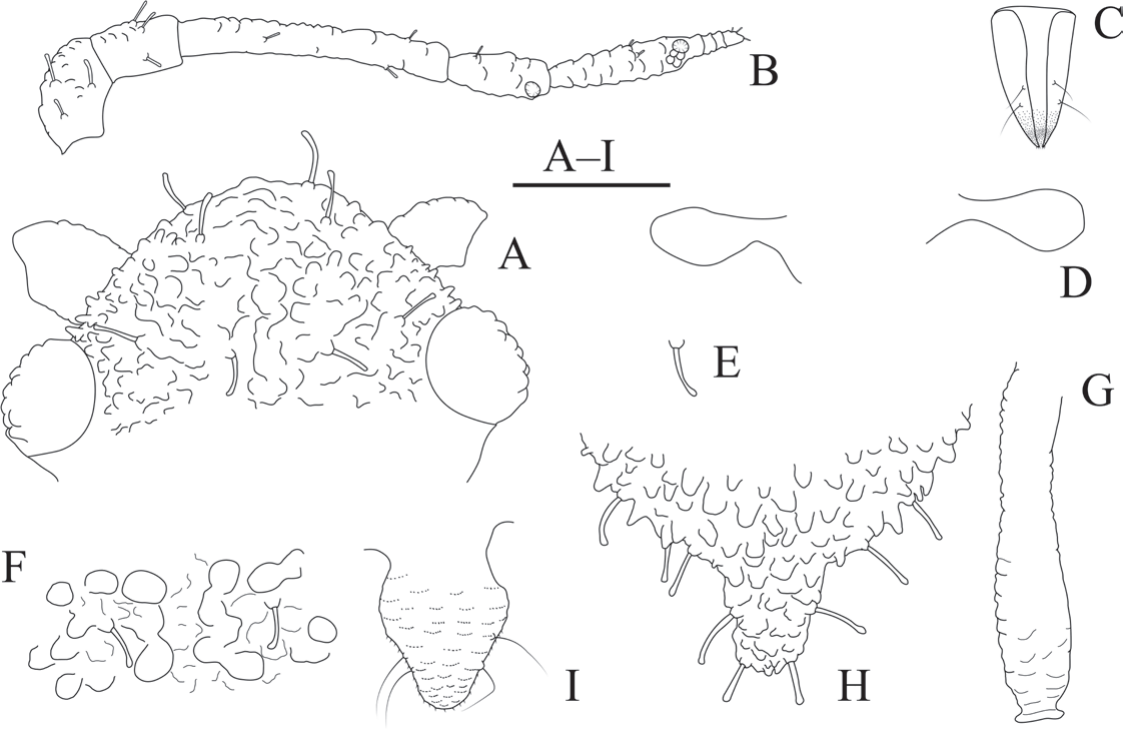
*Cavariellagilgiana* Zhang, Chen, Zhong & Li. Apterous viviparous female: **A** dorsal view of head **B** antenna **C** ultimate rostral segment **D** mesosternal furca **E** marginal seta of abdominal tergite I **F** sculptures of abdominal tergites **G** siphunculus **H** supra-caudal process on abdominal tergite VIII **I** cauda. Scale bar: 0.10 mm.

**Figure 11.**
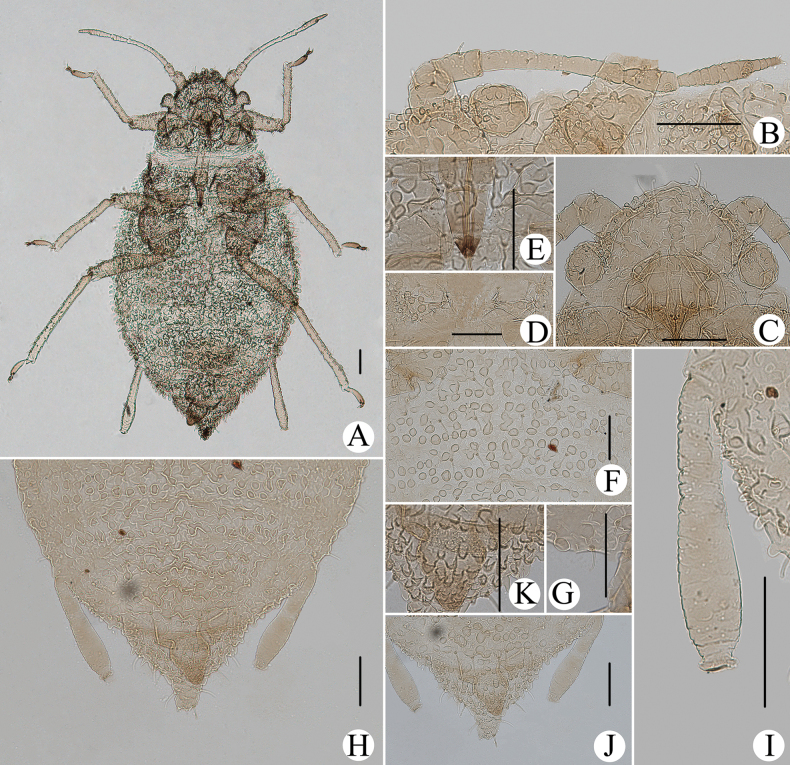
*Cavariellagilgiana* Zhang, Chen, Zhong & Li. Apterous viviparous female: **A** dorsal view of body **B** antenna **C** dorsal view of head **D** mesosternal furca **E** ultimate rostral segment **F** spino-pleural sculptures of abdominal tergites **G** marginal seta of abdominal tergite I **H** abdominal tergites IV–VIII **I** siphunculus **J** supra-caudal process on abdominal tergite VIII **K** cauda. Scale bars: 0.10 mm.

Figs 10 , 11 , 32H , Table 2

Cavariella gilgiana Zhang, Chen, Zhong & Li, 1999: 370.

#### Types examined.

***Holotype***: one ap. viv. fem., **China: Ningxia** (Yinchuan City), 10.VI.1976, No. Y1156-1-1-2, on *Salix*, coll. Unknown. ***Paratypes***: four ap. viv. fems., with the same collection data as holotype (Zhang et al 1999).

#### Other specimens examined.

Five ap. viv. fems. (slides) and one ap. viv. fem. (COI: OP956147), Sichuan (Ganzi City), 15.VI.2021, No. 51429, on *Salix*, coll. T.Y. Liu and S. Xu; six ap. viv. fems., Qinghai (Huangyuan County), 9.VI.1997, No. 11400, on *Salix*, coll. X.L. Chen.

#### Diagnosis.

Body dorsum covered with densely papillate tubercles (Figs 10F, 11F, H); antennae 5-segmented (Figs 10B, 11B), PT short, 0.36–0.40× Ant. Vb; dorsal setae of body long, thick, and capitate (Figs 10E, 11G); ABD TERG VIII produced caudad into triangular spinal supra-caudal process (Figs 10H, 11J); cauda pentagonal, constricted at base and distal part (Figs 10I, 11K) (Zhang et al. 1999).

#### Re-description.

**Apterous viviparous females**: body oval, pale yellowish in life (Fig. 32H).

***Mounted specimens*.** Body pale, unsclerotized; Ant. V, distal part of rostrum, HT II brown, other parts pale in color. Body dorsum covered with densely papillate tubercles (Fig. 11A). See Table 2 for general measurements.

**Table 2.** Morphometric data on species of *Cavariella* (mm).

Parts		*C.gilgiana*	*C.lhasana*		*C.sculptura* sp. nov.	
		Apterous viviparous females (*n* = 5)	Apterous viviparous females (*n* = 3)	Alate viviparous females (*n* = 2)	Apterous viviparous females (*n* = 11)	Alate viviparous female (*n* = 1)
Length (mm)	Body length	1.248–1.466	1.748–2.001	1.702–1.968	1.941–2.333	2.185
	Body width	0.671–0.809	0.883–1.100	0.713–0.786	0.990–1.265	1.012
	Antennae	0.418–0.530	0.701–0.744	1.110–1.177	0.700–0.888	/
	Ant. I	0.054–0.074	0.064–0.071	0.074–0.086	0.076–0.095	0.088
	Ant. II	0.046–0.061	0.054–0.060	0.060–0.063	0.052–0.064	0.068
	Ant. III	0.142–0.205	0.184–0.207	0.384–0.418	0.229–0.310	/
	Ant. IV	0.053–0.074	0.086–0.092	0.152–0.162	0.080–0.128	/
	Ant. V	/	0.089–0.104	0.125–0.149	0.093–0.107	/
	Ant. Vb / Ant. VIb	0.083–0.098	0.083–0.105	0.119–0.130	0.087–0.111	/
	PT	0.032–0.037	0.113–0.135	0.186–0.179	0.103–0.127	/
	URS	0.079–0.092	0.116–0.139	0.111–0.119	0.141–0.151	0.139
	Single arm of mesosternal furca	0.084–0.099	0.161	/	0.147–0.200	/
	Hind femur	0.247–0.291	0.375–0.413	0.453–0.524	0.467–0.536	0.604
	Hind tibia	0.376–0.473	0.651–0.747	0.894–0.970	0.738–0.908	1.118
	HT II	0.086–0.099	0.119–0.130	0.125–0.127	0.115–0.131	0.122
	SIPH	0.227–0.247	0.319–0.366	0.297–0.324	0.354–0.418	0.278
	BW SIPH	0.028–0.033	0.074–0.091	0.055–0.065	0.057–0.093	0.069
	MW SIPH	0.023–0.036	0.049–0.056	0.026–0.043	0.052–0.074	0.047
	SW SIPH	0.039–0.048	0.071–0.084	0.066–0.080	/	/
	DW SIPH	0.027–0.030	0.039–0.044	0.041–0.044	0.041–0.046	0.036
	Cauda	0.103–0.128	0.130–0.182	0.140–0.143	0.125–0.158	0.135
	BW Cauda	0.075–0.099	0.094–0.135	0.088–0.121	0.107–0.146	0.069
	MW Cauda	0.056–0.069	0.078–0.088	0.046–0.047	0.072–0.085	0.047
	Ant. III BD	0.016–0.023	0.027–0.029	0.019–0.023	0.030–0.037	0.02
	Hind tibia MW	0.030–0.036	0.039–0.056	0.033–0.043	0.038–0.048	0.037
	Cephalic setae	0.035–0.043	0.011–0.017	/	0.020–0.024	/
	Setae on Tergite I	0.032–0.038	/	/	0.013–0.026	0.016
	Setae on Tergite VIII	0.033–0.037	0.019–0.023	0.016	0.032–0.045	0.046
	Setae on Ant. I–II	0.016–0.026	/	/	/	/
	Setae on Ant. III	0.006–0.008	0.006–0.008	0.010–0.012	0.009–0.012	/
	Setae on Hind tibia	0.022–0.029	0.022–0.027	0.023–0.028	0.022–0.035	0.031
	supra-caudal process on Tergite VIII	0.090–0.103	0.075–0.136	0.026–0.031	0.063–0.073	/
	BW of supra-caudal process on Tergite VIII	0.103–0.130	0.094–0.120	0.05	0.051–0.083	/
Ratio (times)	Body length / Body width	1.76–1.86	1.82–1.98	2.39–2.50	1.81–2.01	2.16
	Whole antennae / Body	0.33–0.38	0.37–0.42	0.60–0.65	0.33–0.41	/
	Hind femur / Ant. III	1.36–1.74	1.81–2.17	1.18–1.25	1.58–2.10	/
	Hind tibia / Body	0.28–0.34	0.36–0.37	0.49–0.53	0.35–0.43	0.51
	Ant. I / Ant. III	0.34–0.43	0.32–0.35	0.19–0.21	0.28–0.39	/
	Ant. II / Ant. III	0.28–0.33	0.26–0.31	0.15–0.16	0.18–0.27	/
	Ant. IV / Ant. III	0.35–0.44	0.42–0.50	0.36–0.42	0.28–0.49	/
	Ant. V / Ant. III	/	0.46–0.51	0.33–0.36	0.36–0.47	/
	Ant. Vb or Ant. VIb / Ant. III	0.41–0.59	0.45–0.51	0.31	0.28–0.47	/
	PT / Ant. III	0.16–0.23	0.55–0.72	0.43–0.48	0.33–0.55	/
	PT / Ant. Vb or Ant. VIb	0.36–0.40	1.08–1.59	1.38–1.56	1.02–1.25	/
	URS / BW URS	1.46–1.98	1.63–1.87	2.09	2.39–3.33	2.24
	URS / HT II	0.86–0.96	0.94–1.07	0.89–0.94	1.08–1.25	1.14
	Cauda / BW Cauda	1.13–1.42	1.24–1.38	1.16–1.63	0.94–1.45	1.06
	Cauda / MW Cauda	1.70–2.04	1.60–2.07	3.04	1.74–2.08	2.5
	Cephalic setae / Ant. III BD	1.74–2.38	0.41–0.63	/	0.65–0.77	/
	Setae on Tergite I / Ant. III BD	1.39–2.00	/	/	0.55–0.84	/
Ratio (times)	Setae on Tergite VIII / Ant. III BD	1.57–2.31	0.69–0.85	0.84	1.00–1.45	2.3
	Setae on ANT. III / ANT. III BD	0.26–0.42	0.22–0.28	0.52–0.53	0.28–0.38	/
	Setae on hind tibia / Hind tibia MW	0.61–0.83	0.48–0.56	0.65–0.70	0.46–0.78	0.84
	SIPH / Body	0.17–0.19	0.18–0.19	0.17–0.18	0.16–0.20	0.13
	SIPH / Cauda	1.93–2.36	2.01–2.45	2.08–2.31	2.41–2.98	2.06
	SIPH / Ant. III	1.20–1.71	1.54–1.93	0.77–078	1.17–1.78	/
	SIPH / BW SIPH	7.36–8.57	4.02–4.81	4.99–5.40	4.01–7.16	4.03
	SIPH / MW SIPH	6.69–10.43	5.70–7.47	7.54–11.42	5.04–7.59	5.92
	SIPH / SW SIPH	5.04–6.33	3.80–5.15	4.05–4.50	/	/
	SIPH / DW SIPH	7.57–8.93	7.25–9.38	6.75–7.90	7.87–9.95	7.72
	Single arm of Mesosternal furca / Ant. III	0.43–0.63	0.88	/	0.76	/
	supra-caudal process on Tergite VIII / Cauda	0.70–1.00	0.78–0.90	0.19–0.22	0.47	/

***Head*.** Ocular tubercles indistinct. Dorsum of head covered with semicircular and wavy sculptures on median area, marginal area with papillate tubercles (Figs 10A, 11C). Frons convex (Figs 10A, 11C). Dorsal setae of head long, thick, and capitate, with distinct setal tubercles. Head with one pair of cephalic setae, one or two pairs of dorsal setae between antennae, two pairs of dorsal setae between compound eyes arranged transversely. Antennae 5-segmented, Ant. I and II slightly imbricated at inner side, Ant. III–V with slight imbrications (Figs 10B, 11B). Antennal setae long, thick, and blunt or capitate on Ant. I and II, 0.02–0.05 mm, short and blunt on Ant. III–V. Ant. I–V each with 3 or 4, 2 or 3, 2 or 3, 1 or 2, 2 or 3+0 setae; apex of PT with two or three setae. Primary rhinaria ciliated. Rostrum reaching mid-coxae; URS wedge-shaped (Figs 10C, 11E), with two pairs of primary setae, without accessory setae.

***Thorax*.** Thoracic nota with circular and semicircular sculptures on spino-pleural areas, marginal areas with papillate tubercles. Mesosternal furca separated (Figs 10D, 11D). Dorsal setae of thorax long, thick and capitate; pronotum with two pairs of spinal setae, arranged anteriorly and posteriorly, one pair of pleural and one pair of marginal setae; mesonotum with 5–7 spino-pleural setae and two pairs of marginal setae; metanotum with 2–4 spino-pleural setae and two pairs of marginal setae. Legs short. Femora thick and short. Outsides of femora and tibiae imbricated. Setae on legs long, thick and capitate. First tarsal chaetotaxy: 3, 2, 2. Second tarsal segments with imbrications.

***Abdomen*.** Abdominal tergites with circular and semicircular sculptures on spino-pleural areas, marginal areas with papillate tubercles (Figs 10F, 11F, H). ABD TERG VIII produced caudad into triangular spinal supra-caudal process and covered with papillate tubercles, constricted and blunt at distal part, exceeding to the end of cauda (Figs 10H, 11J). Dorsal setae of abdomen long, thick, and capitate (Figs 10E, 11J); abdominal tergites I–V each with two or three pairs of spino-pleural setae and one or two pairs of marginal setae, tergite VI with one pair of spinal and one pair of marginal setae, tergite VII with one pair of spinal, pleural and marginal setae, respectively; tergite VIII with two setae at apex and 2–4 marginal setae of supra-caudal process. Spiracles reniform and open. SIPH long clavate, basal 1/2 cylindrical and then gradually swollen towards apical part but constricted at apex (Figs 10G, 11I); basal part smooth, distal part with imbrications, with flange. Cauda pentagonal (Figs 10I, 11K), constricted at base and distal part, with spinulose imbrications and 2–5 setae. Anal plate semicircular, spinulose, with 8–12 setae. Genital plate transversely oval, with sparse spinules in transverse rows, with two anterior setae and six or seven setae along the posterior margin.

#### Comment.

The species was first described by Zhang et al. (1999), but the description was brief and incomplete. So, a detailed description, character illustration, ecological photographs, and DNA barcoding are supplied in this work.

#### Biology.

The species feeds on young leaves of *Salix* (Fig. 32H).

#### Distribution.

China (Ningxia, Sichuan, Qinghai).

### ﻿ Cavariella japonica

Taxon classificationAnimaliaHemipteraAphididae

(Essig & Kuwana, 1918)

620C315D-C5B2-59D7-A3E7-EB78ADAE27A1

Figs 12 , 33A–C

Siphocoryne japonica Essig & Kuwana, 1918: 66.

#### Specimens examined.

One ap. viv. fem. and one ala. viv. fem. (slides), one ap. viv. fem. (COI: OP956124), **China: Hubei** (Yien County), 30.IV.2016, No. 36809-1-1, on Apiaceae, coll. X.C. Zhu; one ap. viv. fem. and one ala. viv. fem., **Hubei** (Xingdou Mountain), 4.V.2019, No. 45380-1-1, on Apiaceae, coll. X.L. Zhang; three ap. viv. fems., **Hubei** (Xingdou Mountain), 3.V.2019, No. 45369-1-1, on Apiaceae, coll. X.L. Zhang; one ap. viv. fem. and one ala. viv. fem. (slides), one ap. viv. fem. (COI: OP956119), **Sichuan** (Dujiangyan), 11.IV.2014, No. 31362-1-1, on Apiaceae, coll. Y. Wang and X.J. Tang; two ap. viv. fems., **Hubei** (Xingdou Mountain), 3.V.2019, No. 45370-1-1, on Apiaceae, coll. X. L. Zhang; two ap. viv. fems. (slides) and one ap. viv. fem. (COI: OP956136), **Sichuan** (Chengdu City), 24.VIII.2018, No. 44015-1-1, on Apiaceae, coll. Y. Xu and J.F. Ji; two ap. viv. fems. (slides) and one ap. viv. fem. (COI: OP956135), **Sichuan** (Chengdu City), 11.VIII.2018, No. 43839-1-1, on Apiaceae, coll. Y. Xu and J.F. Ji.

#### Diagnosis.

**Figure 12.**
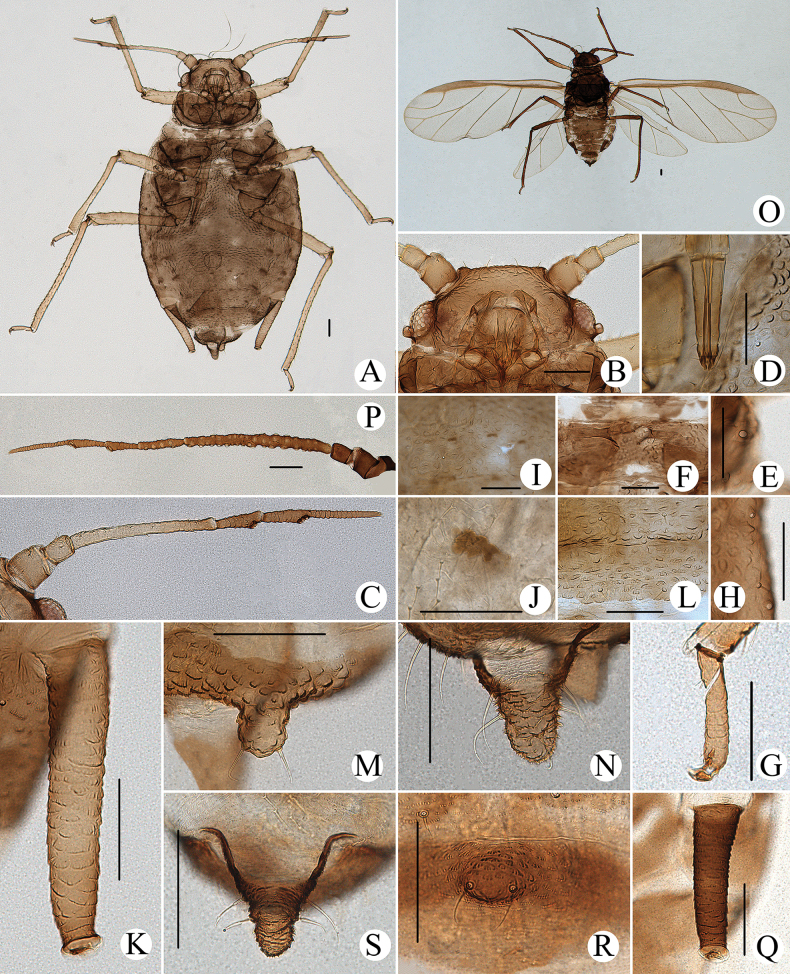
*Cavariellajaponica* (Essig & Kuwana). Apterous viviparous female: **A** dorsal view of body **B** dorsal view of head **C** antenna **D** ultimate rostral segment **E** marginal tubercle of prothorax notum **F** mesosternal furca **G** second hind tarsal segment **H** marginal tubercles of abdominal tergite I–II **I** sculptures of abdominal tergites I–VI **J** muskelplatten **K** siphunculus **L** sculptures of abdominal tergites VII **M** supra-caudal process on abdominal tergite VIII **N** cauda. Alata viviparous female: **O** dorsal view of body **P** antenna **Q** siphunculus **R** supra-caudal process on abdominal tergite VIII **S** cauda. Scale bars: 0.10 mm.

In life, body white, distal part of tibiae and tarsi black, other parts pale, body dorsum sometimes slightly sclerotized (Fig. 33A, B); nymphs white, unsclerotized (Fig. 33C). In mounted specimens, body dorsum covered with oval sculptures and papillate tubercles, sometimes sclerotized and pale brown in color (Fig. 12A); PT 1.41–1.64× Ant. VIb; SIPH cylindrical, tapering, constricted distally, slightly curved outward at the end (Fig. 12K), 0.14–0.18× body length; ABD TERG VIII with short conical supra-caudal (Fig. 12M); Ant. III–V each with 31–38, 4 or 5, 0 or 1 circular secondary rhinaria in alatae (Fig. 12P) (Essig and Kuwana 1918).

#### Comment.

The species resembles *Cavariellanigra*, but differs as follows: body white, dorsum sometimes slightly sclerotized, distal part of tibiae and tarsi black, other parts pale in life; nymphs white, unsclerotized in life (*C.nigra*: body dorsum sclerotized and black, and appendages black in life; nymphs pale yellow, thoracic nota and abdominal tergites each with one pair of spinal and marginal patches); PT 1.41–1.64× Ant. VIb (*C.nigra*: PT 1.17–1.49× Ant. VIb); Ant. III–V each with 31–38, 4 or 5, 0 or 1 circular secondary rhinaria in alatae (*C.nigra*: Ant. III–V each with 51–64, 11 or 12, 1–3 circular secondary rhinaria in alatae).

#### Biology.

Primary host plant: *Salix*, and the aphids feed on young stems (Fig. 33A). Secondary host plant: Apiaceae, and the aphids feed on upper sides of leaves and with ant-attendance (Fig. 33B, C).

#### Distribution.

China (Hubei, Sichuan, Taiwan (Tao 1964)), Japan.

### ﻿ Cavariella konoi

Taxon classificationAnimaliaHemipteraAphididae

Takahashi, 1939

2271F06F-E5DA-524A-8BCA-C6F5D895513F

Figs 13 , 14 , 33D

Cavariella konoi Takahashi, 1939: 117.

#### Specimens examined.

One ap. viv. fem. and one ala. viv. fem. (slides), one ap. viv. fem. (COI: OP956129), **China: Jilin**, 4.VIII.2017, No. 41101-1-1, on *Salix*, coll. H. Long and T.Y. Liu; one ap. viv. fem. and one ala. viv. fem. (slides), one ap. viv. fem. (COI: OP956132), **Jilin**, 8.VIII.2017, No. 41195-1-1, on *Salix*, coll. H. Long and T.Y. Liu.

#### Diagnosis.

**Figure 13.**
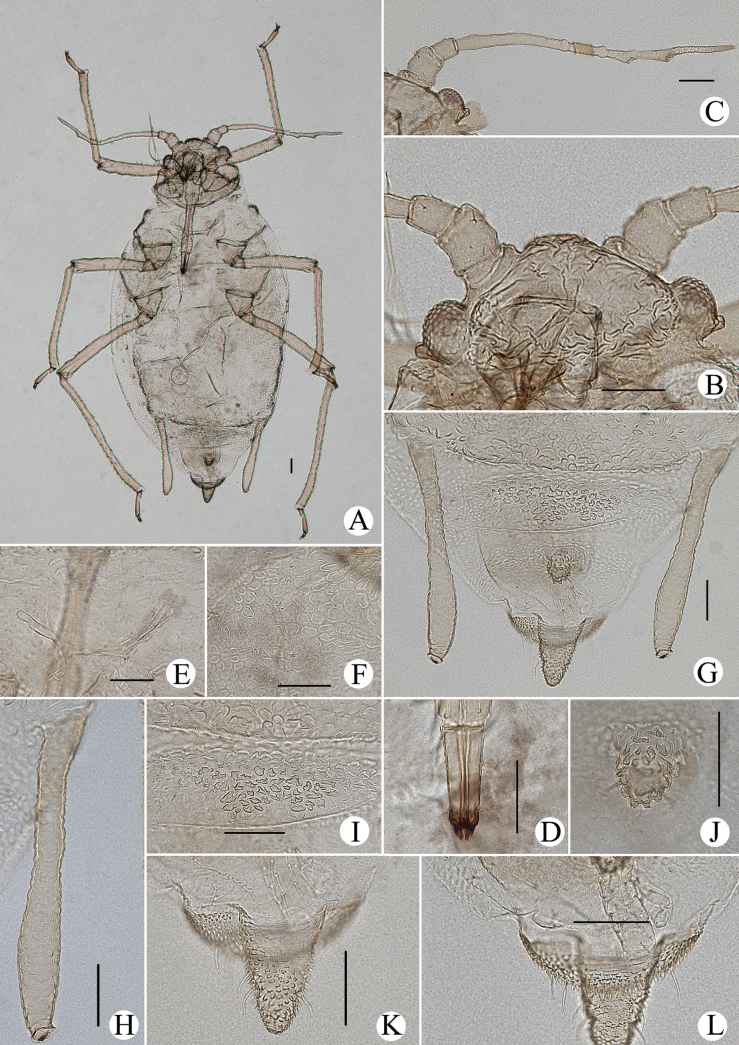
*Cavariellakonoi* Takahashi. Apterous viviparous female: **A** dorsal view of body **B** dorsal view of head **C** antenna **D** ultimate rostral segment **E** mesosternal furca **F** sculptures of abdominal tergites I–VI **G** abdominal tergites VI–VII **H** siphunculus **I** sculptures of abdominal tergite VII **J** supra-caudal process on abdominal tergite VIII **K** cauda **L** anal plate. Scale bars: 0.10 mm.

**Figure 14.**
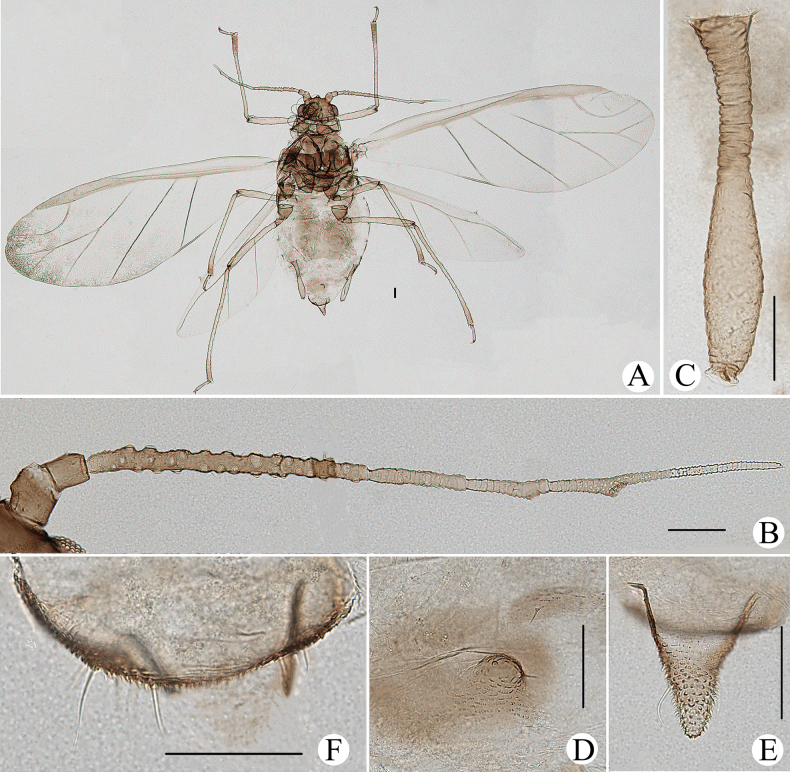
*Cavariellakonoi* Takahashi. Alate viviparous female: **A** dorsal view of body **B** antenna **C** siphunculus **D** supra-caudal process on abdominal tergite VIII **E** cauda **F** anal plate. Scale bars: 0.10 mm.

PT 1.67–1.80× Ant. VIb; URS 1.02–1.08× HT II; SIPH long clavate (Fig. 13H), 0.21–0.22× body length; ABD TERG VIII with short conical supra-caudal (Fig. 13J), 0.04–0.07 mm, as long as basal width; Ant. III and IV each with 28–32, 3 circular secondary rhinaria in alatae (Fig. 14B) (Takahashi 1939).

#### Comment.

*Cavariellakonoi* was first described in China by Zhang et al. (1999), but by checking the specimens, it was established that they were misidentified and should be *Cavariellanigra* Basu, 1964.

#### Biology.

The species was found feeding on the undersides of leaves of *Salix* (Fig. 33D) in China, and it migrate between *Salix* and Apiaceae (Blackman and Eastop 2022).

#### Distribution.

China (Jilin), Canada, Denmark, Finland, Germany, Japan, Mongolia, Norway, Poland, Russia, Sweden, UK, USA.

### ﻿ Cavariella lhasana

Taxon classificationAnimaliaHemipteraAphididae

Zhang, 1981

AD773B4C-EAFE-5280-B087-FB22163911DE

**Figure 15.**
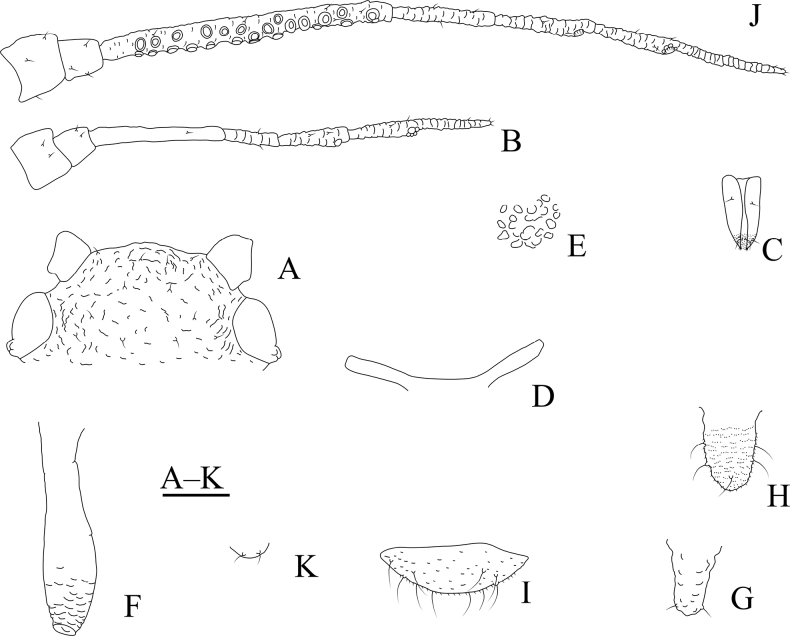
*Cavariellalhasana* Zhang. Apterous viviparous female: **A** dorsal view of head **B** antenna **C** ultimate rostral segment **D** mesosternal furca **E** sculptures of abdominal tergites **F** siphunculus **G** supra-caudal process on abdominal tergite VIII **H** cauda **I** anal plate. Alate viviparous female: **J** antenna **K** supra-caudal process on abdominal tergite VIII. Scale bar: 0.10 mm.

**Figure 16.**
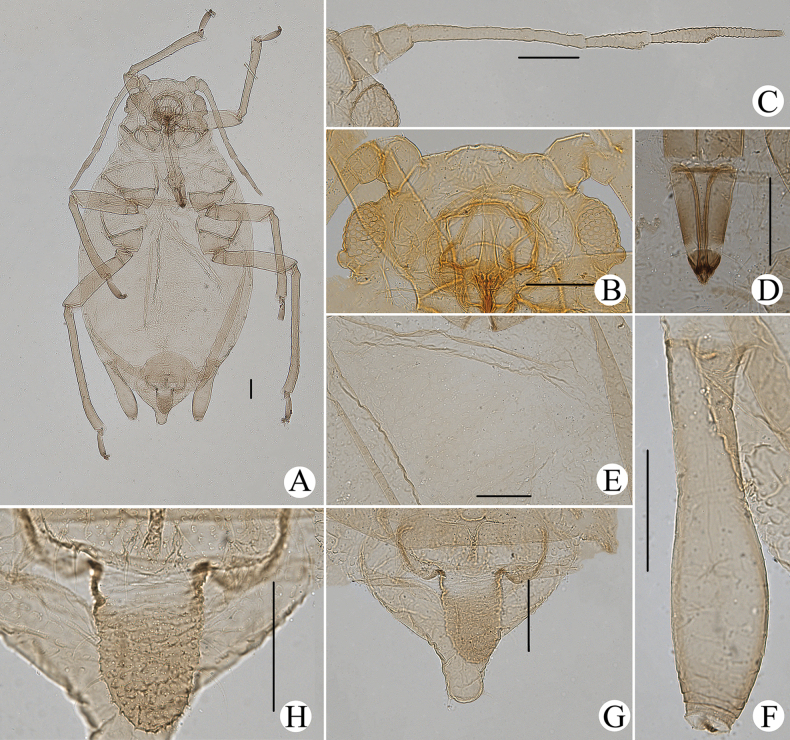
*Cavariellalhasana* Zhang. Apterous viviparous female: **A** dorsal view of body **B** dorsal view of head **C** antenna **D** ultimate rostral segment **E** sculptures of abdominal tergites **F** siphunculus **G** supra-caudal process on abdominal tergite VIII **H** cauda. Scale bars: 0.10 mm.

**Figure 17.**
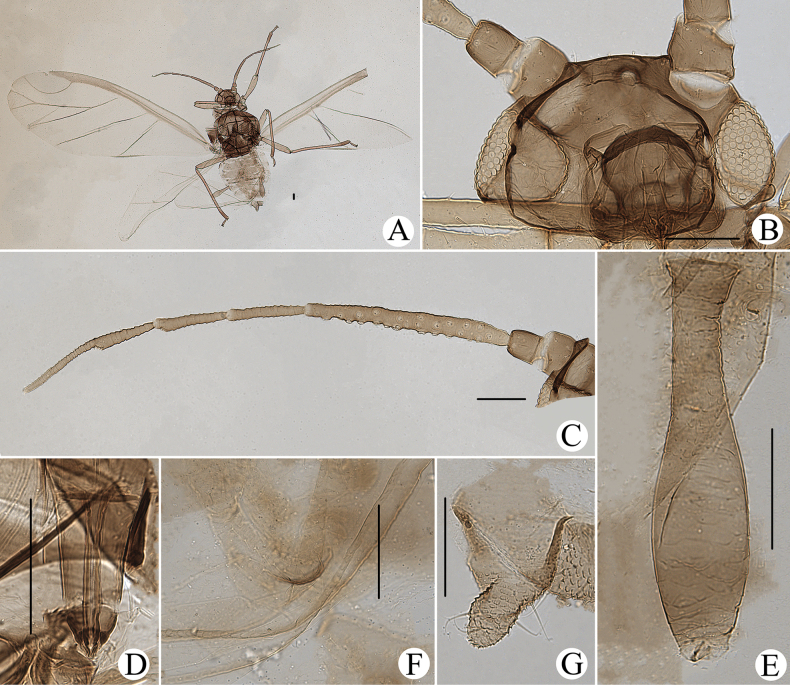
*Cavariellalhasana* Zhang. Alate viviparous female: **A** dorsal view of body **B** dorsal view of head **C** antenna **D** ultimate rostral segment **E** siphunculus **F** supra-caudal process on abdominal tergite VIII **G** cauda. Scale bars: 0.10 mm.

Figs 15 , 16 , 17 , Table 2

Cavariella lhasana Zhang, 1981: 262.

#### Specimens examined.

***Syntypes*.** Three ap. viv. fems. and three ala. viv. fems., **China: Tibet**, 3.IX.1975, No. 6162-1-2, on *Medicago*, coll. Z.Q. Wang (Zhang and Zhong 1981).

#### Diagnosis.

PT 1.08–1.59× Ant. VIb; URS wedge-shaped (Figs 15C, 16D), 1.63–1.87× the base wide, 0.93–1.07× HT II; SIPH clavate, distal part distinctly swollen (Figs 15F, 16F), the length 0.18–0.19× body length, the swollen wide 1.82–1.91× distal width; cauda broadly tongue-shaped (Figs 15H, 16H), 1.24–1.38× basal width (Zhang and Zhong 1981).

#### Re-description.

**Apterous viviparous females**: body oval, green in life.

***Mounted specimens*.** Body pale, unsclerotized; Ant. V and VI, distal part of rostrum, HT II, SIPH, cauda and anal plate pale brown, other parts pale in color (Fig. 16A). See Table 2 for general measurements.

***Head*.** Dorsum of head covered with wrinkles, distinctly at marginal areas. Frons convex with undeveloped antennal tubercles, slightly lower than median frontal tubercles (Figs 15A, 16B). Dorsal setae of head short and blunt. Head with one pair of cephalic setae, one pair of setae at apex of antennal tubercles, two pairs of dorsal setae between antennae, two pairs of dorsal setae between compound eyes arranged transversely. Antennae 6-segmented, Ant. I–III smooth, Ant. IV–VI with slight imbrications (Figs 15B, 16C). Antennal setae short and blunt. Ant. I–VI each with 3, 3 or 4, 3–5, 1 or 2, 2 or 3, 2–4+1 or 2 setae; apex of PT with two or three setae. Primary rhinaria unciliated. Rostrum reaching mid-coxae; URS wedge-shaped (Figs 15C, 16D), with three pairs of primary setae, none or one pair of accessory setae.

***Thorax*.** Thoracic nota with oval and semicircular sculptures, distinctly at marginal areas. Dorsal setae of thorax short and blunt; pronotum with two pairs of spinal setae, arranged anteriorly and posteriorly, one pair of pleural and one pair of marginal setae. Legs short. Distal part of femora and tibiae slightly imbricated. Setae on legs short and blunt. First tarsal chaetotaxy: 3, 3, 3. Second tarsal segments with imbrications.

***Abdomen*.** Abdominal tergites with oval and semicircular sculptures, distinctly at marginal areas. ABD TERG VIII produced caudad into conical spinal supra-caudal process, at least longer than 1/2 of cauda, covered with wavy wrinkles and with two blunt setae at apex (Figs 15G, 16G). Dorsal setae of abdomen short and blunt. Spiracles reniform and open. SIPH clavate, basal 1/2 cylindrical and then distinctly swollen towards apical part but constricted at apex (Figs 15F, 16F), the swollen wide 1.82–1.91× distal wide; basal 2/3 with wrinkles, distal 1/3 imbricated, with flange. Cauda broadly tongue-shaped (Figs 15H, 16H), with spinulose imbrications and five or six setae. Anal plate semicircular (Fig. 15I), spinulose, with 10–16 setae. Genital plate broadly oval, with sparse spinules in transverse rows, with two anterior setae and six or seven setae along the posterior margin.

**Alate viviparous females: *mounted specimens*.** Body long oval; head and thorax black-brown, antennae, legs, distal part of rostrum, SIPH, supra-caudal process, cauda and anal plate brown, other parts pale in color (Fig. 17A). See Table 2 for general measurements.

***Head*.** As in apterous viviparous females except as follows: dorsum of head smooth. Frons convex, antennal tubercles slightly prominent, lower than median frontal tubercle (Fig. 17B). Dorsal setae of head short and pointed. Antennae 6-segmented (Fig. 17C), Ant. I and II smooth, Ant. III–VI with imbrications. Antennal setae short and pointed. Ant. I–VI each with 3, 3, 4, 3, 2 or 3, 2+1 or 2 setae; apex of PT with three setae. Primary rhinaria ciliated. Ant. III with 22–25 circular secondary rhinaria.

***Thorax*.** As in apterous viviparous females except as follows: dorsum of thorax smooth. Legs normal. Setae on legs long and pointed. Fore wing radius bent, media twice-branched, two cubitus; hind wings with one long longitudinal vein and two obliques.

***Abdomen*.** Abdominal tergites I–VII each with one pair of brown marginal sclerites; tergite I with a band at spino-pleural areas, tergites II–V with a brown quadrate sclerite at spino-pleural areas, tergites VI–VIII each with a brown band. Dorsum of abdominal tergites with spinulose imbrications at sclerites, others smooth. Abdominal tergites I–IV each with one pair of small marginal tubercles; ABD TERG VIII with a short warty supra-caudal process (Figs 15K, 17F), with two pointed setae at apex. Dorsal setae of abdomen long and pointed. SIPH clavate, basal 1/2 cylindrical and then distinctly swollen towards apical part but constricted at apex (Fig. 17E); basal 2/3 with wrinkles, distal 1/3 imbricated, with flange. Cauda conical (Fig. 17G), with spinulose imbrications and five setae. Anal plate semicircular, spinulose, with 12–15 setae. Genital plate broadly round, with sparse spinules in transverse rows, with two anterior setae and seven setae along the posterior margin. Others as in apterous viviparous females.

#### Comment.

The species was described by Zhang and Zhong (1981) in China and was considered as synonym of *Cavariellaaegopodii* in Blackman and Eastop (2022). By checking the six syntypes of the species, it is sure that the species resembles *Cavariellaaegopodii* in dorsum sculptures, conical supra-caudal process, the shape of URS, but the species differs from *C.aegopodii* as follows: URS 0.94–1.07× HT II (*C.aegopodii*: URS distinctly shorter than HT II, 0.73–0.83× HT II); PT 1.08–1.59× Ant. VIb (*C.aegopodii*: PT mostly shorter than Ant. VIb); SIPH thick clavate, distal part distinctly swollen, the swollen wide 1.82–1.91× distal wide (*C.aegopodii*: SIPH clavate, the swollen wide 1.37–1.72× distal wide). Due to these differences in major characters, we remove *C.lhasana* from synonymy with *C.aegopodii* and reinstate it as a valid species.

#### Biology.

The species feeds on leaves of *Medicago*.

#### Distribution.

China (Tibet).

### ﻿ Cavariella nigra

Taxon classificationAnimaliaHemipteraAphididae

Basu, 1964

AD1F9435-751C-5945-82B8-1CE3D615F595

Figs 18 , 19 , 34

Cavariella nigra Basu, 1964: 240.

#### Specimens examined.

One ap. viv. fem. and one ala. viv. fem., **China: Sichuan** (Ganzi City), 18.VII.2017, No. 45858-1-1, on *Salix*, coll. J.F. Ji; one ap. viv. fem. and one ap. viv. fem. (COI: OP956126), **Hubei** (Yien County), 3.V.2016, No. 36849-1-1, on Apiaceae, coll. X.C. Zhu; one ap. viv. fem. (slide) and one ap. viv. fem. (COI: OP956134), **Shaanxi** (Ningshan County), 4.VI.2018, No. 43081-1-1, on Apiaceae, coll. H. Long; one ap. viv. fem. and one ala. viv. fem. (slides), one ap. viv. fem. (COI: OP956130), **Jilin**, 5.VIII.2017, No. 41140-1-1, on Apiaceae, coll. T.Y. Liu and H. Long; two ap. viv. fems., **Yunnan**, 25.IX.2020, No. 49354-1-1, on Apiaceae, coll. Y. Xu; two ap. viv. fems., **Tibet** (Nyingchi City), 30.VI.2021, No. 51743-1-1, on *Salix*, coll. Y. Xu; two ap. viv. fems., **Tibet** (Nyingchi City), 26.VI.2021, No. 51699-1-1, on *Salix*, coll. Y. Xu; three ap. viv. fems. and five ala. viv. fems., **Beijing**, 4.VIII.2000, No. 12561, on Apiaceae, coll. G.X. Qiao.

#### Diagnosis.

**Figure 18.**
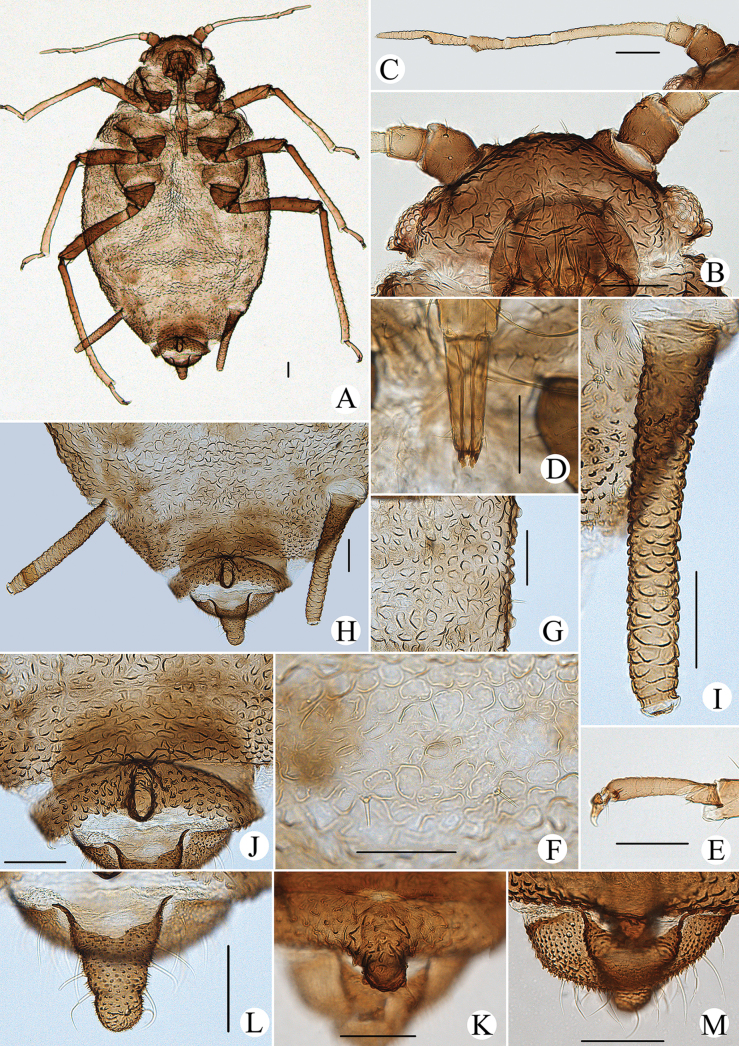
*Cavariellanigra* Basu. Apterous viviparous female: **A** dorsal view of body **B** dorsal view of head **C** antenna **D** ultimate rostral segment **E** second hind tarsal segment **F** spino-pleural sculptures of abdominal tergites I–VI **G** marginal papillate tubercles of abdominal tergite II **H** abdominal tergites V–VIII **I** siphunculus **J** sculptures and papillate tubercles of abdominal tergites VII–VIII **K** supra-caudal process on abdominal tergite VIII **L** cauda **M** anal plate. Scale bars: 0.10 mm.

**Figure 19.**
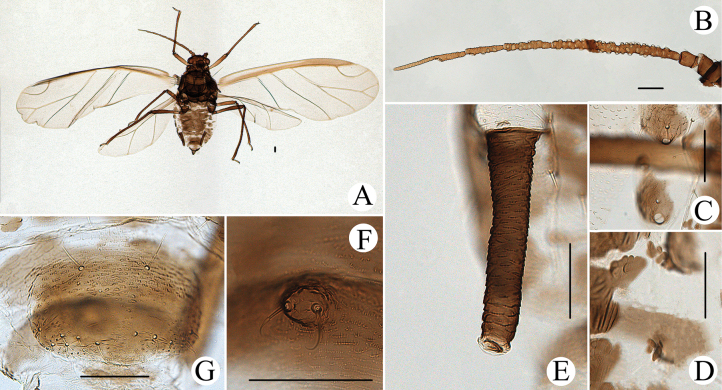
*Cavariellanigra* Basu. Alate viviparous female: **A** dorsal view of body **B** antenna **C** marginal tubercles of abdominal tergite II–III **D** muskelplatten **E** siphunculus **F** supra-caudal process on abdominal tergite VIII **G** genital plate. Scale bars: 0.10 mm.

In life, body dorsum black, venter of abdomen pale yellow, sometimes pink, and appendages black (Fig. 34); nymphs pale yellow, sometimes pink, and appendages black; thoracic nota and abdominal tergites each with one pair of spinal and marginal patches (Fig. 34D). In specimens, Body dorsum sclerotized and uniformly black-brown in mounted specimens (Fig. 18A). PT 1.17–1.49× Ant. VIb. Ant. III–V each with 51–64, 11 or 12, 1–3 circular and produced secondary rhinaria in alatae (Fig. 19B). URS long wedge-shaped (Fig. 18D), reaching hind coxae. ABD TERG VIII with short supra-caudal process (Fig. 18K), 0.05–0.11 mm, 0.84–1.45× basal width. SIPH long cylindrical (Fig. 18I), not swollen, constricted distally, with strongly imbricated.

#### Comment.

The species is recorded in China for the first time. The species resembles *Cavariellajaponica*, but differs as follows: body dorsum sclerotized and black, and appendages uniformly black in life; nymphs pale yellow, thoracic nota and abdominal tergites each with one pair of spinal and marginal patches in life (*C.japonica*: body white, dorsum sometimes slightly sclerotized, distal part of tibiae and tarsi black, other parts pale in life; nymphs white, unsclerotized in life); PT 1.17–1.49× Ant. VIb (*C.japonica*: PT 1.41–1.64× Ant. VIb); Ant. III–V each with 51–64, 11 or 12, 1–3 circular and produced secondary rhinaria in alatae (*C.japonica*: Ant. III–V each with 31–38, 4 or 5, 0 or 1 circular secondary rhinaria in alatae).

#### Biology.

Primary host plants: *Salix*, and the aphids feed on tender tips and with ant attendance (Fig. 34A–C) in China and India (Basu 1964). Secondary host plants are Apiaceae in China, and the aphids feed on young leaves and flowers, with ant attendance (Fig. 34D–F).

#### Distribution.

China (Beijing, Gansu, Hubei, Jilin, Shaanxi, Sichuan, Tibet, Yunnan), India (West Bengal) (Basu 1964).

### ﻿ Cavariella nipponica

Taxon classificationAnimaliaHemipteraAphididae

Takahashi, 1961

FABA365A-9DD9-520F-8ECA-62F130A8F52A

Figs 20 , 35A

Cavariella nipponica Takahashi, 1961: 8.

#### Specimens examined.

One ap. viv. fem., **China: Liaoning**, 29.VI.1984, No. Y4977-1-2, on *Salix*, coll. G.X. Zhang and L.J. Liu (Jiang et al. 2011); two ap. viv. fems. and two ala. viv. fems. (slides), one ap. viv. fem. (COI: OP956118), **Beijing**, 14.V.2014, No. 31000, on *Salix*, coll. Y. Wang, J.J. Tang and F.F. Niu; one ap. viv. fem. and one ala. viv. fem. (slides), one ap. viv. fem. (COI: OP956116), **Sichuan** (Jiuding Mountain), 23.VIII.2013, No. 30077-1-1, host plant unknown, coll. R. Chen; one ap. viv. fem. and one ala. viv. fem., **Beijing**, 4.VIII.2017, No. 41401-1-1, on Apiaceae, coll. G.X. Qiao and X. Yang; two ap. viv. fems. (slides) and one ap. viv. fem. (COI: OP956150), **Sichuan** (Aba City), 22.VI.2021, No. 51630-1-1, on *Salix*, coll. T.Y. Liu and S. Xu; one ap. viv. fem., **Beijing**, 17.VII.2016, No. 37063-1-1, on unknown, coll. R.J. Zhang and S.F Xu.

#### Diagnosis.

**Figure 20.**
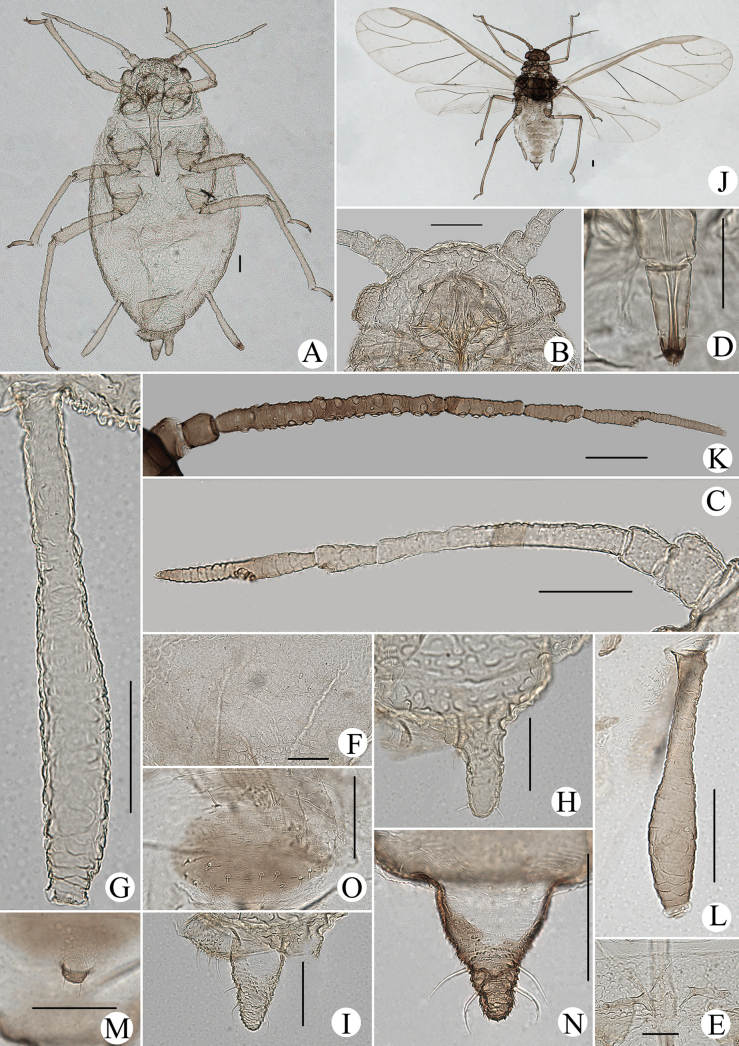
*Cavariellanipponica* Takahashi. Apterous viviparous female: **A** dorsal view of body **B** dorsal view of head **C** antenna **D** ultimate rostral segment **E** mesosternal furca **F** sculptures of abdomen **G** siphunculus **H** supra-caudal process on abdominal tergite VIII **I** cauda. Alate viviparous female: **J** dorsal view of body **K** antenna **L** siphunculus **M** supra-caudal process on abdominal tergite VIII **N** cauda **O** genital plate. Scale bars: 0.10 mm.

ABD TERG VIII with long conical supra-caudal (Fig. 20H), 0.15–0.27 mm, 0.92–1.85× cauda; PT mostly short than Ant. VIb (Fig. 20C); URS wedge-shaped (Fig. 20D), 1.01–1.07× HT II; SIPH long clavate (Fig. 20G), 2.16–2.59× cauda, 0.20–0.22× body length; Ant. III–V each with 26–36, 5 or 6, 1 or 2 circular secondary rhinaria in alatae (Fig. 20K).

#### Comment.

The species has some variations in China, as follows: PT 0.83–1.26× Ant. VIb, in most specimens PT shorter than Ant. VIb, but sometimes PT slightly longer than Ant. VIb (original description: PT 0.70–0.75× Ant. VIb); URS 1.01–1.07× HT II (original description: URS 1.40× HT II) (Takahashi 1961).

#### Biology.

Primary host plant: *Salix*; secondary host plant: Apiaceae including *Anthriscus*, *Angelica*, *Heracleum* (Takahashi 1961). The species feeds on young leaves of the host plant (Fig. 35A).

#### Distribution.

China (Beijing, Hebei, Liaoning, Sichuan), Bhutan, Japan, Russia.

### ﻿ Cavariella pastinacae

Taxon classificationAnimaliaHemipteraAphididae

(Linnaeus, 1758)

220A7092-B186-5700-AE38-2BD6502A6924

Figs 21 , 35B

Aphis pastinacae Linnaeus, 1758: 451.

#### Specimens examined.

One ap. viv. fem. and one ala. viv. fem., **Mongolia**, 11.VII.2010, No. 24728, on Apiaceae, coll. L.Y. Jiang; two ap. viv. fems., **Mongolia**, 15.VII.2010, No. 24756, on Apiaceae, coll. L.Y. Jiang; one ap. viv. fem. (slide) and one ap. viv. fem. (COI: OP956153), **China: Xinjiang**, 2.VII.2022, No. 55639, on Apiaceae, coll. Y. Xu.

#### Diagnosis.

**Figure 21.**
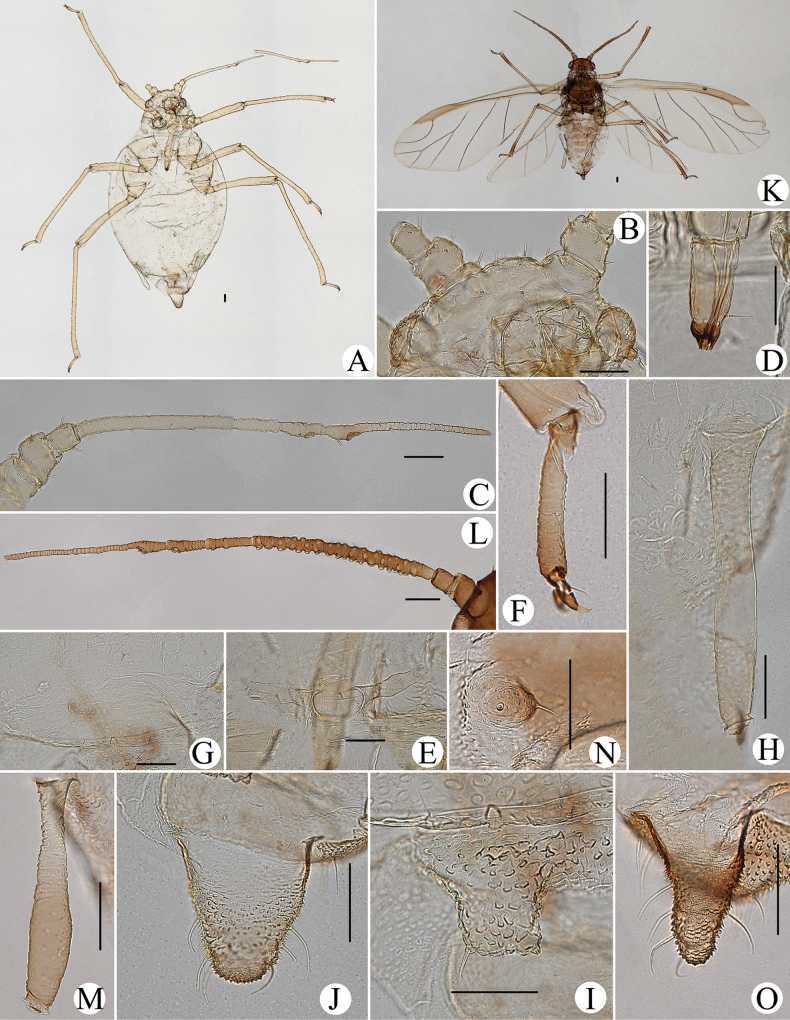
*Cavariellapastinacae* (Linnaeus). Apterous viviparous female: **A** dorsal view of body **B** dorsal view of head **C** antenna **D** ultimate rostral segment **E** mesosternal furca **F** second hind tarsal segment **G** sculptures of abdomen **H** siphunculus **I** supra-caudal process on abdominal tergite VIII **J** cauda. Alate viviparous female: **K** dorsal view of body **L** antenna **M** siphunculus **N** supra-caudal process on abdominal tergite VIII **O** cauda. Scale bars: 0.10 mm.

Antennae 6-segmented (Fig. 21C), PT > 3.46× Ant. VIb; ABD TERG VIII with short rectangular supra-caudal process (Fig. 21I); URS wedge-shaped (Fig. 21D), 1.07–1.15× HT II; SIPH clavate and swollen at middle (Fig. 21H); cauda broadly tongue-shaped (Fig. 21J), with 6–8 setae.

#### Comment.

The species is first recorded in China. The species resembles *Cavariellaangelicae*, but differs as follows: antennae 6-segmented, PT 3.46× Ant. VIb (*C.angelicae*: antennae 5-segmented, PT 1.95–2.39× Ant. Vb); SIPH clavate, swollen at middle and constricted distally, with a subapical annular incision (*C.angelicae*: SIPH cylindrical, no swollen, without annular incision); Ant. III–IV with 46, two circular secondary rhinaria in alatae (*C.angelicae*: only Ant. III with 43 circular secondary rhinaria in alatae).

#### Biology.

Primary host plant *Salix*; secondary host plant Apiaceae including *Heracleum*, *Pastinaca*, *Angelica*, *Carum*, *Chaerophyllum*, *Cicuta*, *Foeniculum* and *Torilis* (Blackman and Eastop 2022). The species feeds on tender tips (Fig. 35B).

#### Distribution.

China (Xinjiang), Argentina, Australia, Europe, Mongolia, North America (Blackman and Eastop 2022).

### ﻿ Cavariella pustula

Taxon classificationAnimaliaHemipteraAphididae

Essig, 1937

238AFD6B-6322-5E55-A309-4763193C0BD8

Figs 22 , 35C

Cavariella pustula Essig, 1937: 46.

#### Specimens examined.

Two ap. viv. fems. and two ala. viv. fems. (slides), one ap. viv. fem. (COI: OP956142), **China: Beijing**, 17.V.2021, No. 50011, on *Salix*, coll. G.X. Qiao and Y. Xu.

#### Diagnosis.

**Figure 22.**
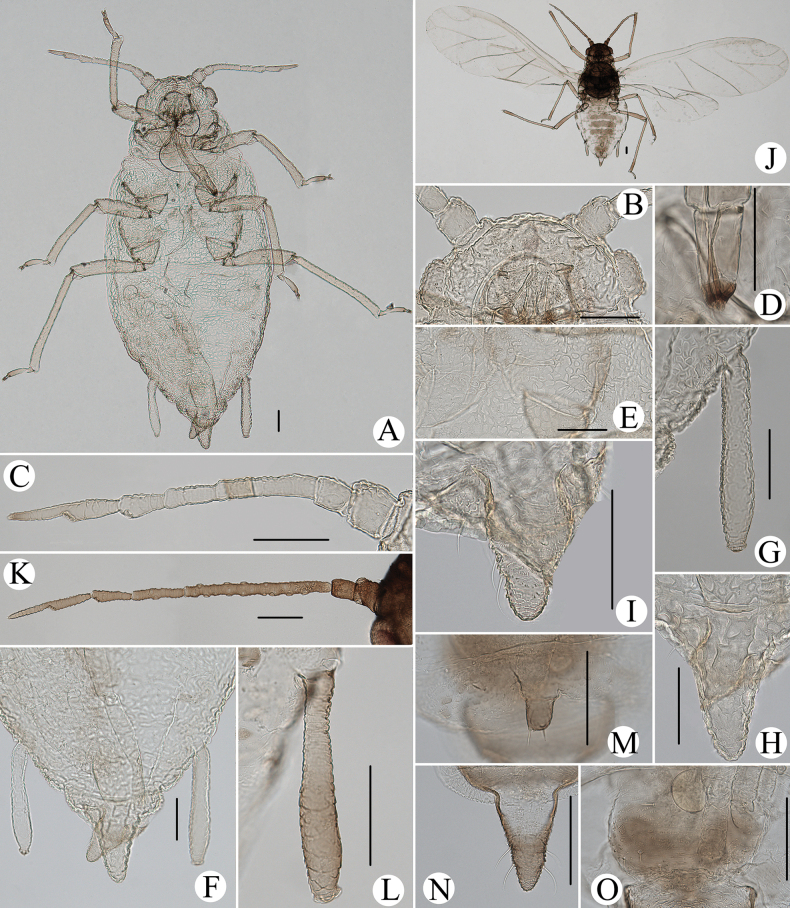
*Cavariellapustula* Essig. Apterous viviparous female: **A** dorsal view of body **B** dorsal view of head **C** antenna **D** ultimate rostral segment **E** sculptures of abdomen **F** abdominal tergites V–VIII **G** siphunculus **H** supra-caudal process on abdominal tergite VIII **I** cauda. Alate viviparous female: **J** dorsal view of body **K** antenna **L** siphunculus **M** supra-caudal process on abdominal tergite VIII **N** cauda **O** genital plate. Scale bars: 0.10 mm.

ABD TERG VIII with hooded supra-caudal completely hiding the cauda from above (Fig. 22H); PT 0.55–0.91× Ant. VIb; URS wedge-shaped (Fig. 22D), 0.94–1.36× HT II; cauda conical and constricted basally, blunt at apex (Fig. 22I), the length 1.45–1.49× basal width (Essig 1937).

#### Comment.

The species is first recorded in China and with some variations in China as follows: Ant. III and IV each with 22, 5 circular secondary rhinaria in alatae, but in USA, Ant. III each with 5–9 circular secondary rhinaria arranged in a row in alatae, and Ant IV without secondary rhinaria (Essig 1937).

The species resembles *Cavariellaaspidaphoides* in ABD TERG VIII hooded supra-caudal process; dorsum of body with circular sculptures; but differs from *C.aspidaphoides* as follows: supra-caudal process only with two setae distally (*C.aspidaphoides*: supra-caudal process with two setae distally and 3–5 short setae marginally); URS with one pair of accessory setae (*C.aspidaphoides*: URS without accessory setae); PT shorter than Ant. VIb (*C.aspidaphoides*: PT longer than Ant. VIb).

#### Biology.

The species feeds on tender tips of *Salix* (Fig. 35C).

#### Distribution.

China (Beijing), Canada, USA.

### ﻿ Cavariella salicicola

Taxon classificationAnimaliaHemipteraAphididae

(Matsumura, 1917)

D6811C09-DBA0-587A-A3CD-C48CEA8FEDF5

Figs 23 , 35D

Nipposiphum salicicola Matsumura 1917: 410.

#### Specimens examined.

One ap. viv. fem. (slide) and one ap. viv. fem. (COI: OP956117), **China: Beijing**, 6.V.2014, No. 30855-1-1, on *Salix*, coll. X.J. Tang and T.T. Xu; one ap. viv. fem. and one ala. viv. fem., **Beijing**, 6.V.2014, No. 30836-1-1, on *Salix*, coll. Y. Wang, X.J Tang and F.F. Niu; two ala. viv. fems., **Hebei**, 12.V.2002, No. 13268-1-1, on *Salix*, coll. G.X. Qiao and H. Liu; one ap. viv. fem., **Beijing**, 26.V.2015, No. 34341-1-1, on *Salix*, coll. X.C. Zhu and Y. Li; two ala. viv. fems., **Qinghai**, 9.VI.1997, No. 11401-1-1, on *Salix*, coll. X.L. Chen (Zhang et al. 1999).

#### Diagnosis.

**Figure 23.**
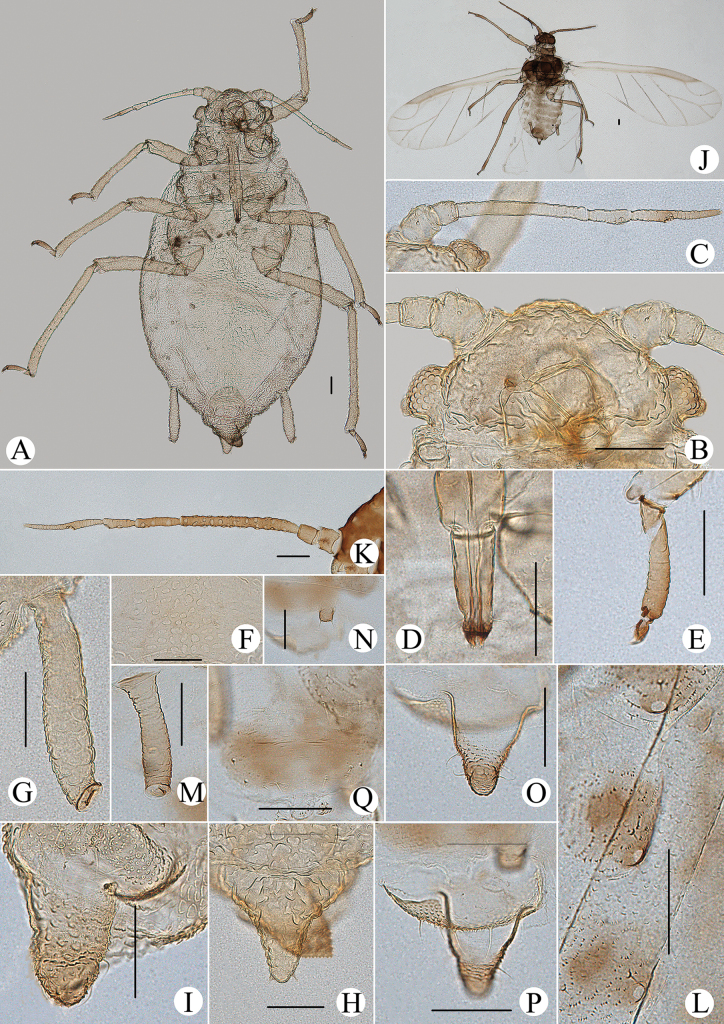
*Cavariellasalicicola* (Matsumura). Apterous viviparous female: **A** dorsal view of body **B** dorsal view of head **C** antenna **D** ultimate rostral segment **E** second hind tarsal segment **F** sculptures of abdomen **G** siphunculus **H** supra-caudal process on abdominal tergite VIII **I** cauda. Alate viviparous female: **J** dorsal view of body **K** antenna **L** marginal tubercles of abdominal tergites II–IV **M** siphunculus **N** supra-caudal process on abdominal tergite VIII **O** cauda **P** anal plate **Q** genital plate. Scale bars: 0.10 mm.

ABD TERG VIII with conical supra-caudal process (Fig. 23H), longer than cauda, 1.60× cauda; SIPH clavate, thick, short, distinctly swollen over most of length, curved outward distally (Fig. 23G), 1.70× cauda; Ant. III–V each with 24–30, 3–7, 0–3 circular secondary rhinaria in alatae (Fig. 23K) (Matsumura 1917; Zhang et al. 1999).

#### Biology.

Primary host plant: *Salix* (Fig. 35D); secondary host plant: Apiaceae (*Apium*, *Oenanthejavanica*, *Cryptotaenia*, *Levisticum*, *Sanicula*, *Sium*) (Blackman and Eastop 2022).

#### Distribution.

China (Beijing, Gansu, Guangdong, Hebei, Henan, Inner Mongolia, Jiangxi, Jiangsu, Jilin, Liaoning, Ningxia, Qinghai, Shaanxi, Shandong, Taiwan, Tianjin, Yunnan, Zhejiang) (Jiang et al. 2011), Japan, Korea, Russia.

### ﻿ Cavariella sculptura

Taxon classificationAnimaliaHemipteraAphididae

Qiao & Xu sp. nov.

55C3C220-2A5C-5E90-BA67-D1235989B7A4

https://zoobank.org/48D595F4-03A0-4996-A26E-F1F38C002DD2

**Figure 24.**
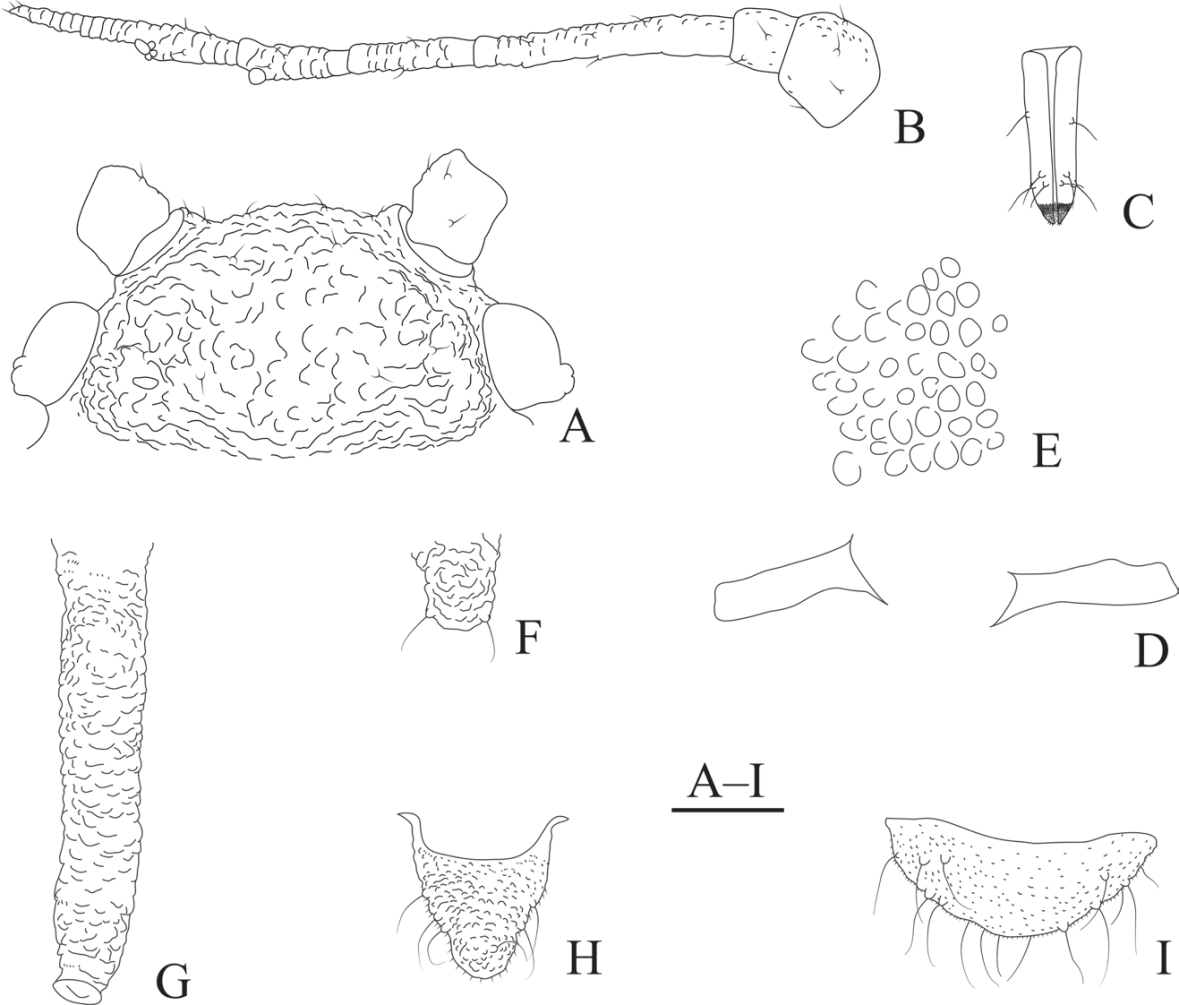
*Cavariellasculptura* Qiao & Xu, sp. nov. Apterous viviparous female: **A** dorsal view of head **B** antenna **C** ultimate rostral segment **D** mesosternal furca **E** spino-pleural sculptures of abdominal tergites I–VI **F** supra-caudal process on abdominal tergite VIII **G** siphunculus **H** cauda **I** anal plate. Scale bar: 0.10 mm.

**Figure 25.**
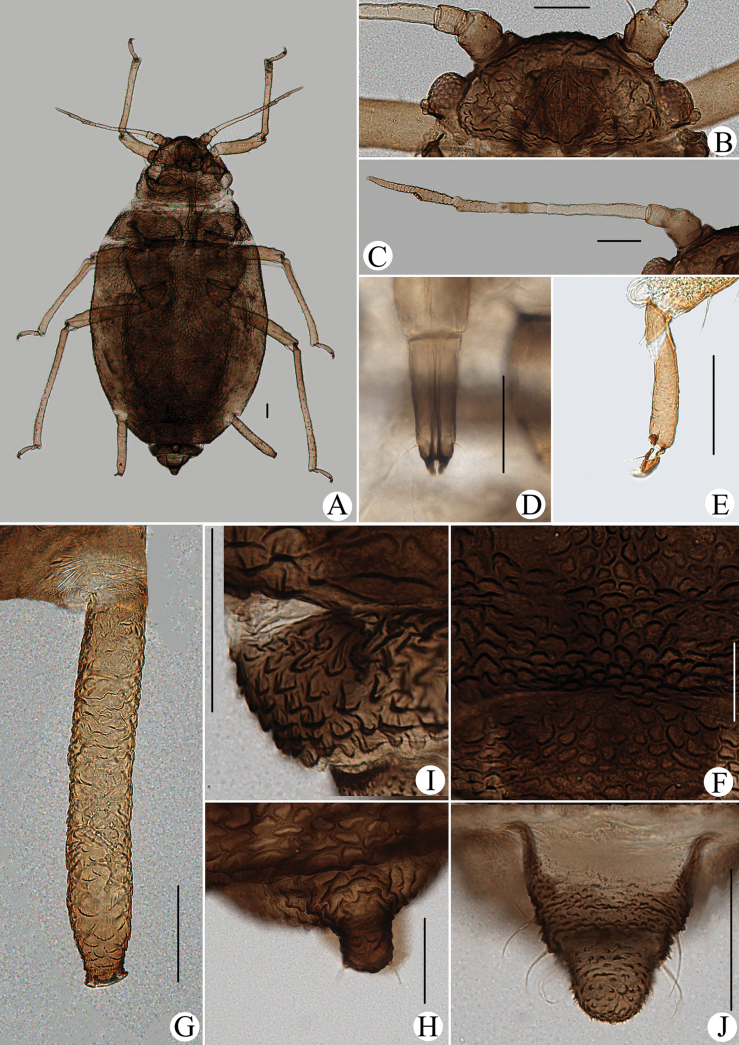
*Cavariellasculptura* Qiao & Xu, sp. nov. Apterous viviparous female: **A** dorsal view of body **B** dorsal view of head **C** antenna **D** ultimate rostral segment **E** second hind tarsal segment **F** sculptures of abdominal tergites I–VI **G** siphunculus **H** supra-caudal process on abdominal tergite VIII **I** marginal papillate tubercles of abdominal tergite VIII **J** cauda. Scale bars: 0.10 mm.

**Figure 26.**
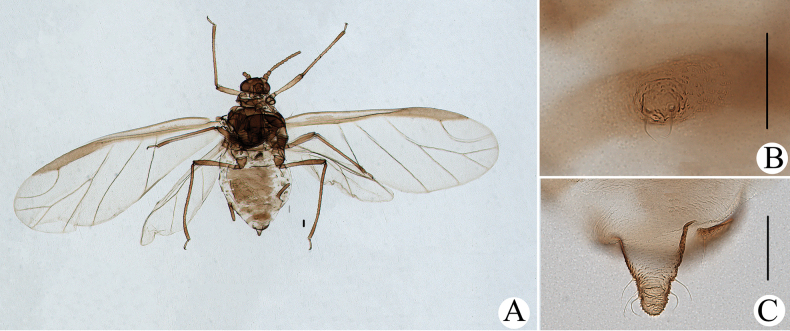
*Cavariellasculptura* Qiao & Xu, sp. nov. Alate viviparous female: **A** dorsal view of body **B** supra-caudal process on abdominal tergite VIII **C** cauda. Scale bars: 0.10 mm.

Figs 24 , 25 , 26 , 36 , Table 2

#### Types examined.

***Holotype***: one ap. viv. fem., **China: Hubei** (Xingdou Mountain), 4.V.2019, No. 45394-1-1-2, on *Torilisscabra* (Thunb.) DC., coll. X.L. Zhang. ***Paratypes***: one ap. viv. fem. (slide) and one ap. viv. fem. (COI: OP956138), with the same collection data as holotype; two ap. viv. fems., **Hubei** (Yien County), 30.IV.2019, No. 45340-1-1, on *Torilisscabra* (Thunb.) DC., coll. X.L. Zhang; one ala. viv. fem. and one ap. viv. fem., **Hubei** (Yien County), 3.V.2016, No. 36855-1-1, on *Torilisscabra* (Thunb.) DC., coll. X.C. Zhu; two ap. viv. fems., **Hubei** (Xingdou Mountain), 3.V.2019, No. 45372-1-1, on *Torilisscabra* (Thunb.) DC., coll. X.L. Zhang; one ap. viv. fem. (slide) and one ap. viv. fem. (COI: OP956127), **Hubei** (Yien County), 3.V.2016, No. 36853-1-1, on *Cryptotaeniajaponica* Hassk., coll. X.C. Zhu; two ap. viv. fems., **Shaanxi** (Ningshan County), 10.VI.2018, No. 43306-1-1, on Apiaceae, coll. H. Long; one ap. viv. fem. (slide) and one ap. viv. fem. (COI: OP956125), **Hubei** (Yien County), 2.V.2016, No. 36840-1-1, on *Torilisscabra* (Thunb.) DC., coll. X.C. Zhu (NHMUK); one ap. viv. fem., **Hubei** (Yien County), 3.V.2016, No. 36848-1-1, on *Torilisscabra* (Thunb.) DC., coll. X.C. Zhu; one ap. viv. fem., **Hubei** (Yien County), 5.V.2016, No. 36902-1-1, on *Torilisscabra* (Thunb.) DC., coll. X.C. Zhu.

#### Diagnosis.

In life, body dorsum of body sclerotized, black and ridged, turtle-shaped, venter of abdomen flat and pink, appendages black distally, other parts pale in color (Fig. 36); in mounted specimens body dorsum sclerotized and inconsistently black, spinal area dark brown and pleuro-marginal area pale brown in color (Fig. 25A); body dorsum with densely semicircular and circular sculptures (Fig. 25A); URS long wedge-shaped (Figs 24C, 25D); abdominal tergites VIII with short cylindrical supra-caudal process (Figs 24F, 25H); SIPH cylindrical and constricted, curved outward distally (Figs 24G, 25G).

#### Description.

**Apterous viviparous females**: body oval, dorsum of body sclerotized, black and ridged, turtle-shaped, venter of abdomen flat and pink, appendages black distally, other parts pale in life (Fig. 36). Nymphs: body pick or orange in color, appendages black distally, thoracic nota and abdominal tergites each with one pair of black spinal and marginal patches, other parts pale in life (Fig. 36B).

***Mounted specimens*.** Body dorsum sclerotized and inconsistently black, spinal area dark brown and pleuro-marginal area pale brown in color (Fig. 25A). Ant. I, II, V, and VI brown, other parts pale brown in color. Legs and SIPH pale brown; cauda and anal plate dark brown in color. Body dorsum covered with densely circular or semicircular sculptures which more developed on spino-pleural areas. See Table 2 for general measurements.

***Head*.** Head dorsum densely covered with wavy sculptures. Frons convex, antennal tubercles slightly prominent, as high as median frontal tubercle (Figs 24A, 25B). Dorsal setae of head short and pointed. Head with one pair of cephalic setae, one or two pairs of setae at apex of antennal tubercles, one or two pairs of dorsal setae between antennae, two pairs of dorsal setae between compound eyes arranged transversely. Antennae 6-segmented (Figs 24B, 25C), 5-segmented occasionally, Ant. I and II slightly imbricated at inner side, Ant. III–VI with imbrications. Antennal setae short and pointed. Ant. I–VI each with 3–5, 3 or 4, 3–9, 2 or 3, 2–5, 2–4+1 or 2 setae; apex of PT with two or three setae. Primary rhinaria unciliated. Rostrum exceeding mid-coxae; URS long wedge-shaped (Figs 24C, 25D), with three pairs of primary setae, one pair of accessory setae.

***Thorax*.** Thoracic nota with semicircular and irregular circular sculptures on spino-pleural areas, marginal areas with semicircular sculptures which sparser than spino-pleural areas. Pronotum mostly with one pair of small marginal tubercles. Dorsal setae of thorax short and pointed; pronotum with two pairs of spinal setae, arranged anteriorly and posteriorly, one pair of pleural and one pair of marginal setae; mesonotum with 6–11 spino-pleural setae and two pairs of marginal setae; metanotum with 4–6 spino-pleural setae and two pairs of marginal setae. Legs normal. Distal part of femora with oval and imbricated sculptures; distal part of tibiae imbricated. Setae on legs short and pointed. First tarsal chaetotaxy: 3, 3, 3. Second tarsal segments with imbrications.

***Abdomen*.** Abdominal tergites I–VI with densely circular sculptures on spino-pleural areas (Figs 24E, 25F) and sclerotized dark brown, marginal areas with sparsely semicircular sculptures; abdominal tergite VII with circular sculptures on spino-pleural areas, marginal and posterior with papillate tubercles; ABD TERG VIII with densely papillate tubercles (Fig. 25I). Abdominal tergites I–IV mostly each with one pair of small marginal tubercles; ABD TERG VIII with short cylindrical supra-caudal process covered with semicircular sculptures (Fig. 25H), 0.06–0.07 mm, 1.11–1.43× basal width. Dorsal setae of abdomen short and pointed; abdominal tergites I–V each with two pairs of spinal setae, one pair of pleural and one pair of marginal setae, tergite VI with one pair of spinal, pleural, and marginal setae respectively, tergite VII with one pair of spinal setae; tergite VIII with two spinal setae at apex of supra-caudal process. Spiracles reniform and open. SIPH cylindrical and constricted at apex, curved outward distally (Figs 24G, 25G); strongly imbricated, with flange. Cauda conical, blunt, and constricted distally (Figs 24H, 25J), with spinulose imbrications and five or six setae. Anal plate semicircular, spinulose (Fig. 24I), with 9–22 setae. Genital plate broadly round, with sparse spinules in transverse rows, with two anterior setae and 8–10 setae along the posterior margin.

**Alate viviparous females**: body long oval; head and thorax black, abdomen pink, abdominal tergites with a quadrate patch in life (Fig. 36C).

***Mounted specimens*.** Head and thorax black-brown, antennae, legs, distal part of rostrum, SIPH, cauda and anal plate brown, other parts pale in color (Fig. 26A). See Table 2 for general measurements.

***Head*.** As in apterous viviparous females except as follows: dorsum of head smooth. Frons convex, antennal tubercles slightly prominent, not higher than frontal tubercle. Antennae lost (in this specimen). Rostrum reaching mid-coxae.

***Thorax*.** As in apterous viviparous females except as follows: dorsum of thorax smooth. Legs normal. Fore wings radius bent, media twice branched, two cubitus; hind wings with single long longitudinal vein and two obliques.

***Abdomen*.** Abdominal tergites I–V each with one pair of marginal sclerites; tergite I with scattered small sclerites at spino-pleural area, tergite II with a brown band at spino-pleural area, tergites III–VI with a brown quadrate sclerite at spino-pleural areas, tergites VII and VIII each with a across brown band (Fig. 26A). Abdominal tergites with spinulose imbrications at sclerites, others smooth. Abdominal tergites I–IV each with one pair of small marginal tubercles; ABD TERG VIII with a short warty supra-caudal process and with two long and pointed setae at apex (Fig. 26B). Cauda conical (Fig. 26C), with spinulose imbrications and five setae. Anal plate semicircular, spinulose, with 16 setae. Genital plate broadly round, with sparse spinules in transverse rows, with two anterior setae and ten setae along the posterior margin. Others as in apterous viviparous females.

#### Etymology.

The new species is named for its sculptures on the dorsum of body.

#### Comment.

The species resembles *Cavariellanigra* in body dorsum sclerotized; URS long wedge-shaped; short supra-caudal process, but differ from it as follows: dorsum of body ridged, turtle-shaped, venter of abdomen flat and pink in color, appendages black distally, other parts pale in life; in nymphs: body pink or orange in color, appendages black distally, other parts pale (*C.nigra*: dorsum of body flat, venter of abdomen yellowish white in color; appendages black wholly in life; in nymphs: body yellowish white in color, appendages black wholly in life); body dorsum sclerotized and inconsistently black in specimens, spinal area of dorsum dark brown and pleuro-marginal ones of dorsum pale brown in color; the SIPH pale brown and same as pleuro-marginal area of dorsum in color (*C.nigra*: body dorsum sclerotized and uniformly black in specimens, the SIPH black brown and same as dorsum of body); marginal area of body with semicircular sculptures (*C.nigra*: marginal area of body with papillate tubercles).

#### Biology.

The species feeds on young stems and leaves of Apiaceae (*Torilis*, *Cryptotaenia*), with ant attendance (Fig. 36).

#### Distribution.

China (Hubei, Shaanxi).

### Subgenus ﻿ Cavariellinepicauda

Taxon classificationAnimaliaHemipteraAphididae

Ivanovskaja, 1980

8873F948-EA00-5B6E-8CCE-9B3512C61362

Cavariellinepicauda Ivanovskaja, 1980: 79.

#### Diagnosis.

The species of the subgenus mostly feed on Apiaceae and are covered with wax in life (Fig. 37). In collected specimens, abdominal tergites I–IV often have circular marginal tubercles; ABD TERG VIII with indistinct supra-caudal process, shorter than Ant. II; rostrum long, sometimes reaching abdominal tergite IV, URS elongated wedge-shaped; cauda tongue-shaped, with > 8 setae; SPHI long cylindrical, with distinct flange, not swollen.

**Figure 27.**
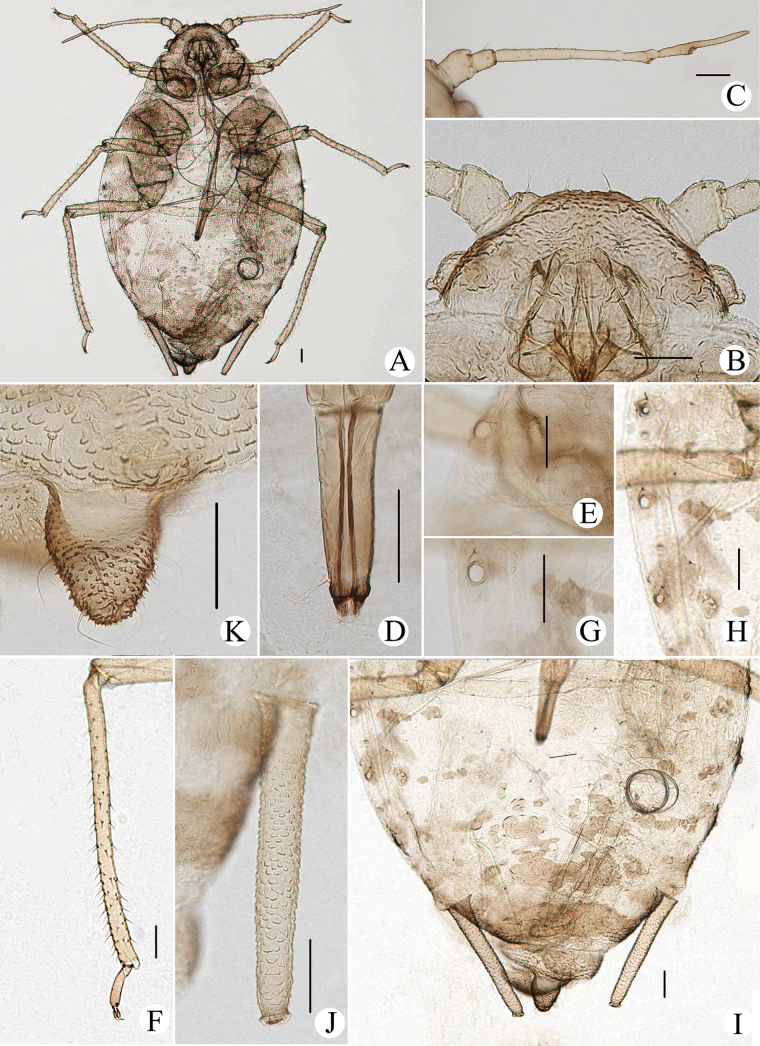
Cavariella (Cavariellinepicauda) cicutisucta Qiao. Apterous viviparous female: **A** dorsal view of body **B** dorsal view of head **C** antenna **D** ultimate rostral segment **E** marginal tubercle of prothorax notum **F** hind tibiae **G** marginal tubercle of abdominal tergite I **H** marginal tubercles of abdominal tergites I–III **I** abdominal tergites II–VIII **J** siphunculus **K** cauda. Scale bars: 0.10 mm.

#### Comments.

The subgenus contains five species, with only two species distributed in China. *Cavariellalargispiracula* Zhang, Chen, Zhong & Li, 1999 is considered as a junior synonym of *Cavariellasapporoensis* Takahashi, 1961. *Cavariellahidaensis* Takahashi, 1961 should be removed to *Elatobium* Mordvilko, 1914 based morphological and molecular data.

### ﻿ Cavariella (Cavariellinepicauda) cicutisucta

Taxon classificationAnimaliaHemipteraAphididae

Qiao, 2005

62AC48A5-2E71-5B2D-AB4E-D087E3416990

Figs 27 , 28 , 37A

Cavariella (Cavariellinepicauda) cicutisucta Qiao in Qiao et al. 2005: 331.

#### Types examined.

***Holotype***: one ap. viv. fem., **China: Shanxi**, 18.VII.2000, No. Y8507-1-1-1, on *Cicutavirosa*, coll. L.J. Zhang.

#### Other specimens examined.

three ap. viv. fems. and one ala. viv. fem. (slides), one ap. viv. fem. (COI: OP956123), **Guizhou**, 27.VII.2014, No. 33648, on Apiaceae, coll. F.F. Niu and Y.Q. Li.

#### Diagnosis.

Body white, covered with wax in life (Fig. 37A); thoracic nota and abdominal tergites I–IV each with one pair of circular marginal tubercles (Fig. 27E, G, H); thoracic nota and abdominal tergites I–IV each with one or two pairs of pale brown marginal sclerites, tergites V and VI often with small brown spino-pleural sclerites, tergites VII and VIII each with a brown sclerotic band (Fig. 27I); dorsal setae short and blunt; rostrum reaching abdominal tergite III, URS elongate wedge-shaped, with five or six secondary setae (Fig. 27D); cauda tongue-shaped (Fig. 27K), with 8–13 setae (Qiao et al. 2005).

**Figure 28.**
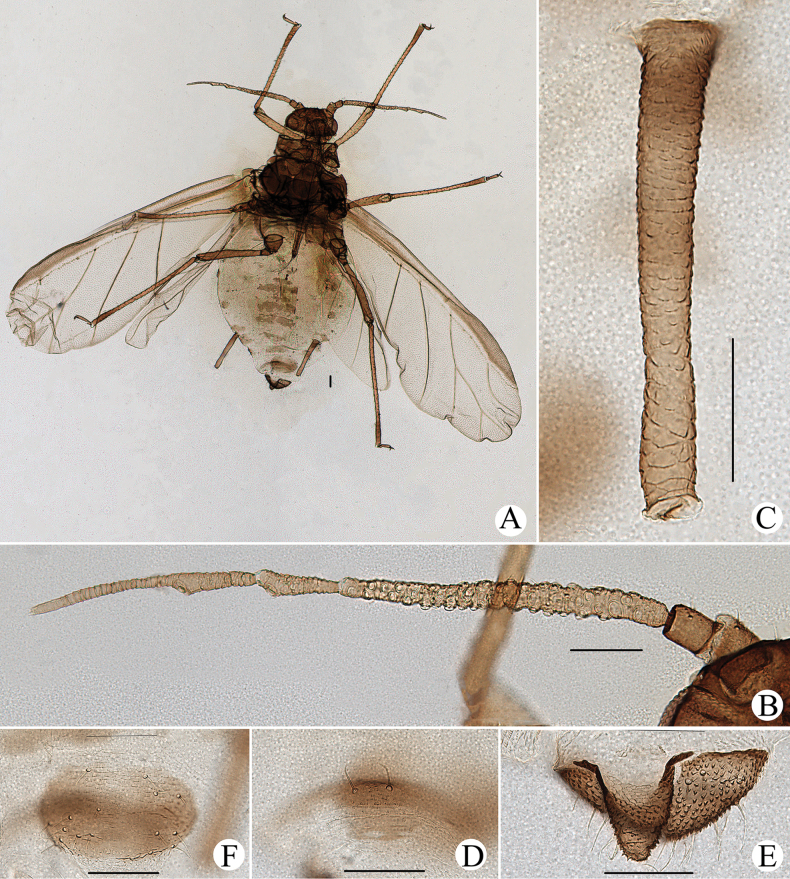
Cavariella (Cavariellinepicauda) cicutisucta Qiao. Alate viviparous female: **A** dorsal view of body **B** antenna **C** siphunculus **D** supra-caudal process on abdominal tergite VIII **E** cauda and anal plate **F** genital plate. Scale bars: 0.10 mm.

#### Comment.

The species resembles *Cavariellasapporoensis*, but the main differences between the two species are as follows: abdominal tergites I–IV sclerotized, tergites VII and VIII each with a brown sclerotic band (*C.sapporoensis*: only abdominal tergites VII and VIII sometimes with a brown sclerotic band); dorsal setae short and blunt (*C.sapporoensis*: dorsal setae very long and pointed, curved distally).

#### Biology.

The species feeds on undersides of leaves near the roots of Apiaceae (*Cicutavirosa*) and is with ant attendance (Fig. 37A) (Qiao et al. 2005).

#### Distribution.

China (Guizhou, Shanxi).

### ﻿ Cavariella (Cavariellinepicauda) sapporoensis

Taxon classificationAnimaliaHemipteraAphididae

Takahashi, 1961

638ADECA-34C2-5D78-B0A2-9114E46B8977

**Figure 29.**
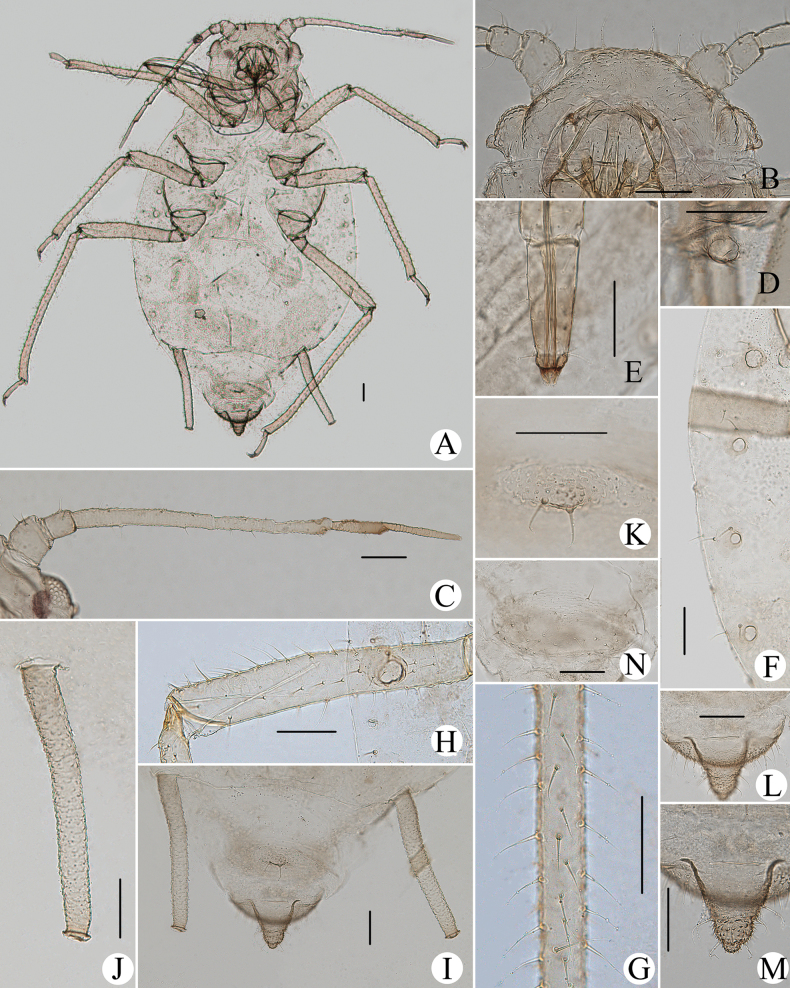
Cavariella (Cavariellinepicauda) sapporoensis Takahashi. Apterous viviparous female: **A** dorsal view of body **B** dorsal view of head **C** antenna **D** marginal tubercle of prothorax notum **E** ultimate rostral segment **F** marginal tubercles of abdominal tubercles I–IV **G** the setae of hind tibiae **H** the setae of hind femur **I** abdominal tergites VI–VIII **J** siphunculus **K** supra-caudal process on abdominal tergite VIII **L** anal plate **M** cauda **N** genital plate. Scale bars: 0.10 mm.

**Figure 30.**
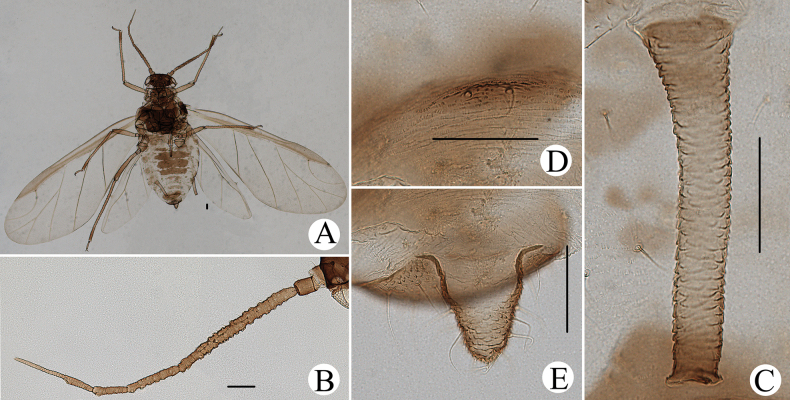
Cavariella (Cavariellinepicauda) sapporoensis Takahashi. Alate viviparous female: **A** dorsal view of body **B** antenna **C** siphunculus **D** supra-caudal process on abdominal tergite VIII **E** cauda. Scale bars: 0.10 mm.

**Figure 31.**
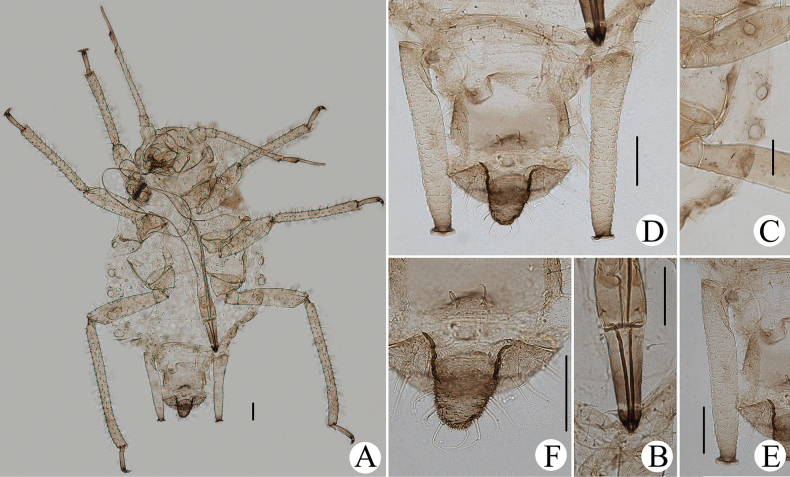
*Cavariellalargispiracula* Zhang, Chen, Zhong & Li, syn. nov. Apterous viviparous female: **A** dorsal view of body **B** ultimate rostral segment **C** marginal tubercles I–IV **D** abdominal tergites VI–VIII **E** siphunculus **F** cauda. Scale bars: 0.10 mm.

Figs 29 , 30 , 31 , 37B–D

Cavariella sapporoensis Takahashi, 1961: 9.

#### Types examined.

***Holotype*** and ***paratypes*** of *Cavariellalargispiracula* Zhang, Chen, Zhong & Li, 1999: two ap. viv. fems., **China: Gansu** (Yuzhong County), 1.VIII.1986, No. 8587-1-2, on Apiaceae, coll. G.X. Zhang, J.H. Li and T.S. Zhong; seventeen apterous oviparous females, **Gansu** (Minxian County), 22.X.1986, No. 8785-1-1, on *Salix*, coll. G.X. Zhang and T. S. Zhong (Zhang et al. 1999).

#### Other specimens examined.

Two ala. viv. fems., **Beijing**, 15.VIII.2017, No. 42056-1-1, on Apiaceae, coll. H. Long; one ap. viv. fem. and one ala. viv. fem. (slides), one ap. viv. fem. (COI: OP956131), **Jilin**, 8.VIII.2017, No. 41184-1-1, on Apiaceae, coll. H. Long and T.Y. Liu; one ap. viv. fem. and one ala. viv. fem. (slides), one ap. viv. fem. (COI: OP956141), **Beijing**, 24.VI.2019, No. 45522-1-1, on Apiaceae, coll. H. Long; one ap. viv. fem. and one ala. viv. fem., **Beijing**, 15.VIII.2017, No. 42057-1-1, on Apiaceae, coll. H. Long; one ap. viv. fem. (slide) and one ap. viv. fem. (COI: OP956140), **Beijing**, 24.VI.2019, No. 45517-1-1, on Apiaceae, coll. H. Long; one ap. viv. fem. (slide) and one ap. viv. fem. (COI: OP956133), **Beijing**, 15.VIII.2017, No. 42064-1-1, on Apiaceae, coll. H. Long; one ap. viv. fem. (slide) and one ap. viv. fem. (COI: OP956139), **Beijing**, 15.VIII.2017, No. 45505-1-1, on Apiaceae, coll. H. Long.

#### Diagnosis.

Pronotum and abdominal tergites I–IV each with large and circular marginal tubercles, larger than spiracles (Fig. 29D, F); setae of legs very long, pointed, dense, and curved apexes (Fig. 29G, H), the setae on femora 0.57–0.78× of widest width, the setae on tibiae 0.97–1.39× of mid-width; cauda tongue-shaped, with 9–16 long and pointed setae (Fig. 29M) (Takahashi 1961).

#### Comment.

After researching the holotype of *Cavariellalargispiracula* Zhang, Chen, Zhong & Li, 1999, we found the species is in fact without large circular spiracles, which are in fact marginal tubercles and with normal reniform spiracles (Fig. 31A, C). In *C.largispiracula*, thoracic nota and abdominal tergites I–IV each with one pair of large marginal tubercles (Fig. 31C); abdominal tergites VIII with a slightly swollen supra-caudal process (Fig. 31D); cauda tongue-shaped (Fig. 31F); setae of legs very long and pointed (Zhang et al. 1999). Therefore, *Cavariellalargispiracula* Zhang, Chen, Zhong & Li, 1999 is considered as a junior synonym of *Cavariellasapporoensis* Takahashi, 1961.

**Figure 32.**
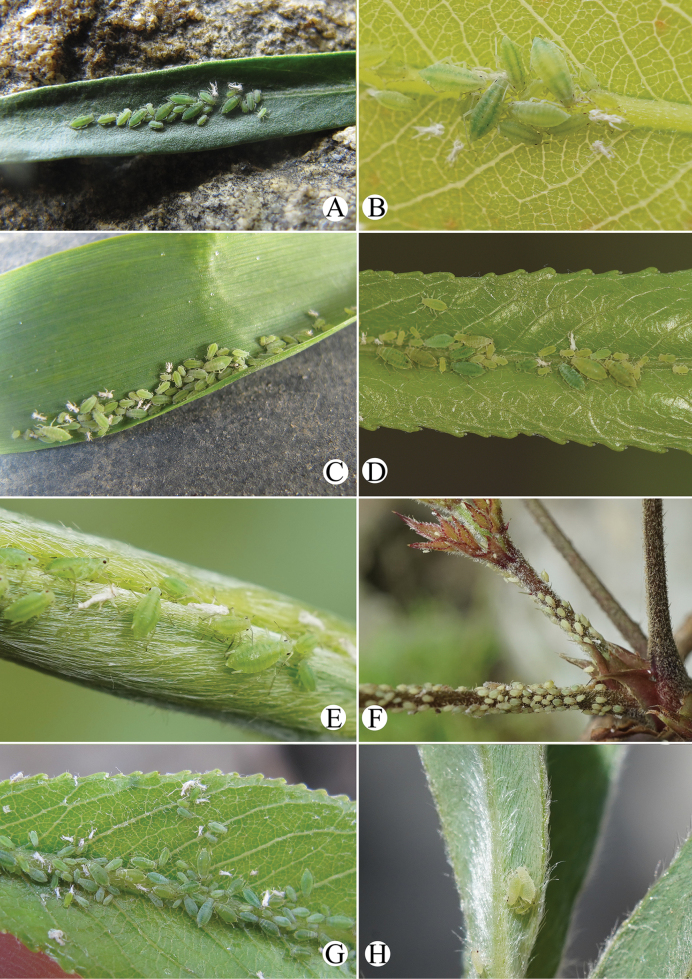
The ecological habitat photographs of *Cavariella* in the field **A, B** the apterae and nymphs of *Cavariellaaquatica* (Gillette & Bragg) feeding on the leaves of *Salix* along main vein **C** the apterae and nymphs of *Cavariellaaquatica* feeding on the leaves of Poaceae**D** the apterae and nymphs of *Cavariellaaegopodii* (Scopoli) feeding on the leaves of *Salix* along main vein **E** the apterae and nymphs of *Cavariellaangelicae* (Matsumura) feeding on the tender leaves of *Salix***F** the apterae and nymphs of *Cavariellaaraliae* Takahashi feeding on tender tips of *Aralia***G** the apterae and nymphs of *Cavariellabhutanensis* Chakrabarti & Das feeding on the leaves of *Salix* along main vein **H** the apterae of *Cavariellagilgiana* Zhang, Chen, Zhong & Li feeding on the leaves of *Salix*.

**Figure 33.**
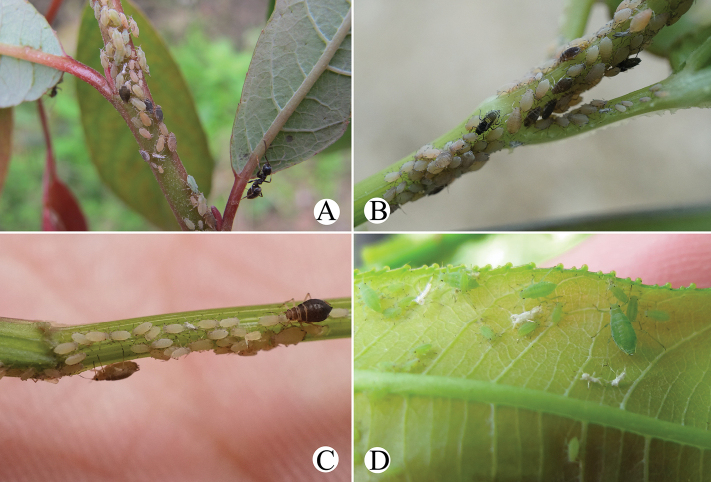
The ecological habitat photographs of *Cavariella* in field **A** the apterae and nymphs of *Cavariellajaponica* (Essig & Kuwana) feeding on tender stem of *Salix***B, C** the apterae and nymphs of *Cavariellajaponica* feeding on tender stem of Apiaceae**D** the apterae and nymphs of *Cavariellakonoi* Takahashi feeding on the leaves of *Salix*.

The species resembles *Cavariellaheraclei* Takahashi, 1961, but differs from it as follows: setae of legs long, pointed, dense, and curved apexes (*C.heraclei*: setae of legs short, blunt, sparse); cephalic setae long and pointed, 1.31–2.74× Ant. IIIBD (*C.heraclei*: cephalic setae short and blunt, 0.50× Ant. IIIBD); setae of Ant. III long and pointed, 0.86–1.24× Ant. IIIBD (*C.heraclei*: setae of Ant. III short, 0.50× Ant. IIIBD) (Takahashi 1961).

**Figure 34.**
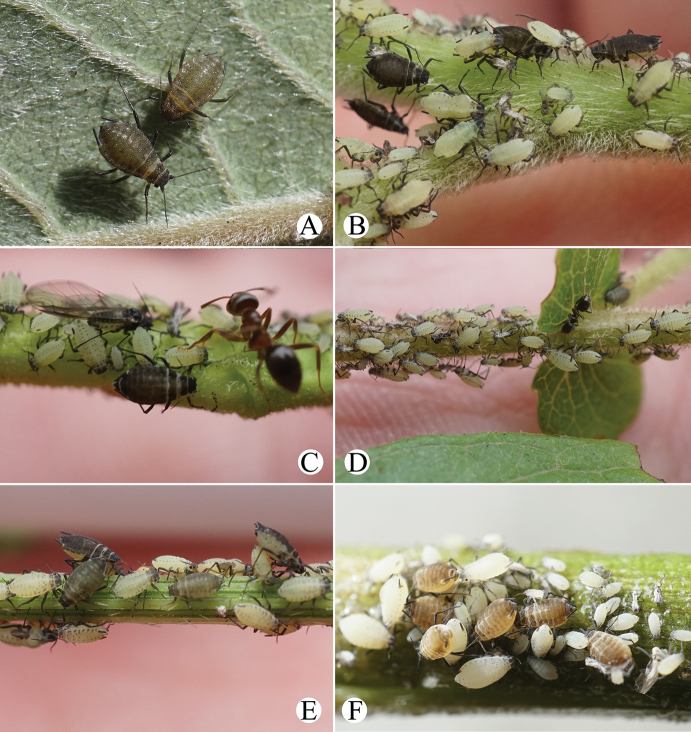
The ecological habitat photographs of *Cavariellanigra* Basu in field **A** two apterae feeding on underside leaves of *Salix* in spring **B** the apterae and nymphs feeding on tender stem of *Salix* in summer **C** the species feeding on *Salix* with ant- attendance **D** the nymphs feeding on tender stem of *Salix* in summer **E, F** the species feeding on Apiaceae.

**Figure 35.**
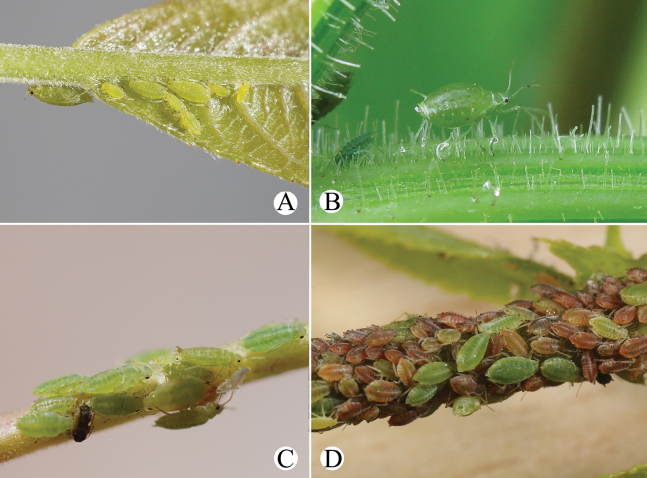
The ecological habitat photographs of *Cavariella* in field. **A** the apterae and nymphs of *Cavariellanipponica* Takahashi feeding on the underside leaves of *Salix***B** one aptera of *Cavariellapastinacae* (Linnaeus) feeding on tender stem of Apiaceae**C** the apterae and nymphs of *Cavariellapustula* Essig feeding on tender stem of *Salix***D** the apterae and nymphs of *Cavariellasalicicola* (Matsumura) feeding on tender stem of *Salix*.

**Figure 36.**
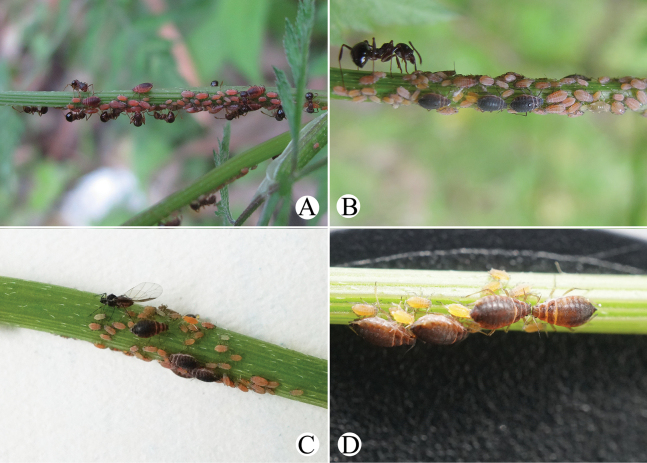
The ecological habitat photographs of *Cavariellasculptura* Qiao & Xu, sp. nov. in field **A, B** the apterae and nymphs feeding on tender stems of Apiaceae (*Torilisscabra*) with ant-attendance **C** one alate, apterae and nymphs feeding on tender stem of Apiaceae (*Torilisscabra*) **D** the apterae and nymphs feeding on tender stem of Apiaceae.

#### Biology.

**Figure 37.**
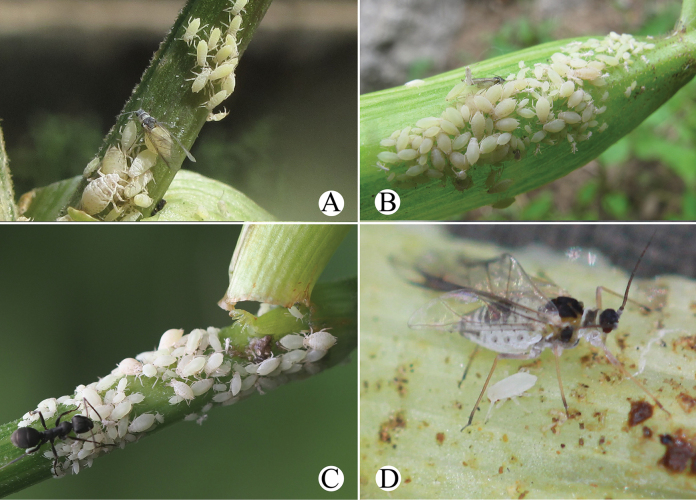
The ecological photos of Cavariella (Cavariellinepicauda) in field **A** one alate, apterae and nymphs of *Cavariellacicutisucta* Qiao feeding on tender stem of Apiaceae**B** the apterae and nymphs of *Cavariellasapporoensis* Takahashi feeding on tender stem of Apiaceae C the apterae and nymphs of *Cavariellasapporoensis* feeding on tender stem of Apiaceae with ant-attendance **D** one alate of *Cavariellasapporoensis* feeding on tender stem of Apiaceae.

The species is usually collected from Apiaceae and feeds on the undersides of leaves with ant attendance (Fig. 37B–D). In China, the apterous oviparous female of the species was found feeding on the leaves of *Salix* in October. So, the species may alternate host between *Salix* and Apiaceae.

#### Distribution.

China (Beijing, Gansu, Jilin), Japan.

### ﻿Keys to the species of *Cavariella* in China

#### ﻿Key to apterae

**Table:** 

1	Siphunculus clavate, without flange, obliquely truncated at tip; cauda long conical, with 7 or 8 setae	***C.aquatica***
–	Siphunculus clavate or cylindrical, with distinct flange; cauda conical or tongue-shaped, with 4–8 or > 8 setae	**2**
2	ABD TERG VIII with slightly swollen supra-caudal process, wart-shaped; cauda tongue-shaped, with > 8 setae	**3**
–	ABD TERG VIII with distinct supra-caudal process, conical; cauda conical, with 4–6 setae	**4**
3	Dorsal setae of body short and blunt; setae on legs short and blunt	***C.cicutisucta***
–	Dorsal setae of body very long and pointed, curved distally; setae on legs long, pointed, and dense	***C.sapporoensis***
4	ABD TERG VIII with very long conical supra-process, pointed apex, much beyond cauda	***C.araliae***
–	ABD TERG VIII with conical supra-caudal, blunt apex, at most a little longer than cauda	**5**
5	SIPH cylindrical, constricted at apex and curved outward distally	**6**
–	SIPH clavate, distinctly swollen	**9**
6	Antennae 5-segmented, PT 1.95–2.39× Ant. Vb; dorsum of body unsclerotized, pale	***C.angelicae***
–	Antennae 6-segmented, occasionally 5-segmented, PT at most 1.50× Ant. VIb; dorsum of body sclerotized, at least spinal of body sclerotized pale brown	**7**
7	Body dorsum of body sclerotized, black and ridged, turtle-shaped in life; in specimens body dorsum sclerotized and inconsistently black, spinal of dorsum dark brown, pleuro-marginal of dorsum and SIPH pale brown; marginal of body with densely semicircular and circular sculptures	***C.sculptura* sp. nov.**
–	Body dorsum of body sclerotized or unsclerotized in life, normal swollen in life; in specimens body dorsum sclerotized consistently black, tergites and SIPH black sometimes unsclerotized; marginal of body with papillate tubercles	**8**
8	Body dorsum sclerotized and black, and appendages uniformly black in life; PT 1.17–1.49× Ant. VIb	***C.nigra***
–	Body white, dorsum sometimes slightly sclerotized, distal part of tibiae and tarsi black, other parts pale in life; PT 1.41–1.64× Ant. VIb	***C.japonica***
9	Antennae 5-segmented, PT 0.36–0.40× Ant. Vb; dorsal setae long, thick, and capitate; body dorsum covered with densely papillate tubercles	***C.gilgiana***
–	Antennae 6-segmented, PT as least longer than 0.5× of Ant. VIb; dorsal setae short and thin; body dorsum covered with circular, semicircular, and wavy sculptures	**10**
10	Abdominal VIII with short conical or rectangular supra-caudal process, the length slightly longer than basal width, not reaching the base of cauda	**11**
–	Abdominal VIII with conical supra-caudal process, the length longer than basal width, at least reaching 1/2 of cauda	**12**
11	ABD TERG VIII with short conical supra-caudal process; PT 1.67–1.80× Ant. VIb; SIPH long clavate, 3.10× cauda	***C.konoi***
–	ABD TERG VIII with rectangular supra-caudal process; PT 3.46× Ant. VIb; SIPH long clavate, 2.63–2.69× cauda	***C.pastinacae***
12	SIPH short clavate, thick, swollen over most of length, 1.70× cauda	***C.salicicola***
–	SIPH long clavate, only distal part swollen, > 2.00× cauda	**13**
13	ABD TERG VIII with hood-shaped supra-caudal, completely covered cauda; PT 0.55–0.91× Ant. VIb	***C.pustula***
–	ABD TERG VIII with conical supra-caudal, thinner than cauda; PT mostly longer than Ant. VIb	**14**
14	PT mostly shorter than Ant. VIb	***C.nipponica***
–	PT as long as or longer than Ant. VIb	**15**
15	URS shorter than HT II, without accessory setae	***C.aegopodii***
–	URS longer than HT II, at least with one pair of accessory setae	**16**
16	SIPH 4.02–4.81× basal width, distal part distinctly swollen, the swollen width 1.82–1.91× distal width; URS wedge-shaped	***C.lhasana***
–	SIPH 5.37–7.69× basal width, distal part swollen, the swollen width 1.40–1.80× distal width; URS long wedge-shaped	***C.bhutanensis***

#### ﻿Key to alatae

**Table:** 

1	Siphunculus clavate, obliquely truncated at tip, without flange	***C.aquatica***
–	Siphunculus clavate or cylindrical, with distinct flange	**2**
2	ABD TERG VIII with indistinct supra-caudal process; cauda tongue-shaped, with > 8 setae	**3**
–	ABD TERG VIII with distinct supra-caudal process, wart-shaped; cauda conical, with 4–6 setae	**4**
3	Dorsal setae of body short and blunt	***C.cicutisucta***
–	Dorsal setae of body very long and pointed, curved distally	***C.sapporoensis***
4	ABD TERG VIII with long and thin conical supra-process	***C.araliae***
–	ABD TERG VIII with wart-shaped supra-caudal, as long as basal with	**5**
5	SIPH cylindrical, not swollen	**6**
–	SIPH clavate, distinctly swollen	**9**
6	Dorsum of body unsclerotized, pale; PT 1.95–2.39× Ant. Vb	***C.angelicae***
–	Dorsum of body strongly sclerotized; PT at most 1.84× Ant.VIb	**7**
7	Ant. III with at most 40 secondary rhinaria; PT 1.56–1.84× Ant. VIb	***C.japonica***
–	Ant. III with > 50 secondary rhinaria; PT at most 1.51× Ant. VIb	**8**
8	Body dorsum of body strongly sclerotized, black; PT 1.14–1.46× Ant. VIb	***C.sculptura* sp. nov.**
–	Body dorsum sclerotized, brown; PT 1.02–1.25× Ant. VIb	***C.nigra***
9	PT 0.39× Ant. VIb	***C.gilgiana***
–	PT at least as long as VIb	**10**
10	Only Ant. III with secondary rhinaria	**11**
–	Ant. III–IV with secondary rhinaria, sometimes Ant. V with secondary rhinaria	**13**
11	URS shorter than HT II, without accessory setae	***C.aegopodii***
–	URS longer than HT II, at least with one pair of accessory setae	**12**
12	SIPH thick clavate, length 4.99–5.40× the basal width, the swollen width 1.95× distal width	***C.lhasana***
–	SIPH long clavate, length 5.48–6.30× the basal width, the swollen width 1.24–1.79× distal width	***C.bhutanensis***
13	SIPH short clavate, swollen over most of length, 1.40× cauda	***C.salicicola***
–	SIPH long clavate, only distal part swollen, > 2.00× cauda	**14**
14	PT 0.83× VIb	***C.pustula***
–	PT at least as long as VIb	**15**
15	PT 4.31× VIb; Ant. III–IV each with secondary rhinaria 46, 2	***C.pastinacae***
–	PT at most 3.00× VIb; Ant. III with secondary rhinaria at most 30	**16**
16	PT 2.11–2.57× VIb; Ant. III–IV each with secondary rhinaria 28–32, 2	***C.konoi***
–	PT 1.11–1.30× VIb; Ant. III–V each with secondary rhinaria 26–36, 5 or 6, 1 or 2	***C.nipponica***

### ﻿Updated key to aphids feeding on *Angelica* based on Blackman and Eastop (2022)

**Table:** 

12	Tergum pigmented, and with nodulose ornamentation; cauda with 5–8 setae	**12a**
–	Tergum pale, smooth; cauda with 9–16 setae	**13**
12a	Body dorsum of body sclerotized, black and ridged, turtle-shaped in life; in specimens body dorsum sclerotized and inconsistently black, spinal of dorsum dark brown, pleuro-marginal of dorsum and SIPH pale brown; marginal of body with densely semicircular and circular sculptures	***C.sculptura* sp. nov.**
–	Body dorsum of body sclerotized or unsclerotized in life, normal swollen in life; in specimens body dorsum sclerotized consistently black, tergites and SIPH black sometimes unsclerotized; marginal of body with papillate tubercles	**12b**
12b	Body dorsum sclerotized and black, and appendages uniformly black in life; PT 1.17–1.49× Ant. VIb; Ant. III–V each with 51–64, 11 or 12, 1–3 circular and produced secondary rhinaria in alatae	***C.nigra***
–	Body white, dorsum sometimes slightly sclerotized, distal part of tibiae and tarsi black, other parts pale in life; PT 1.41–1.64× Ant. VIb; Ant. III–V each with 31–38, 4 or 5, 0 or 1 circular secondary rhinaria in alatae	***C.japonica***
13	R IV+V 1.2–1.3 × HT II; dorsal setae long and pointed, curved distally	***C.sapporoensis***
–	R IV+V 1.6–2.0 × HT II; dorsal setae short and blunt	***C.heraclei***

### ﻿DNA barcoding

**Figure 38.**
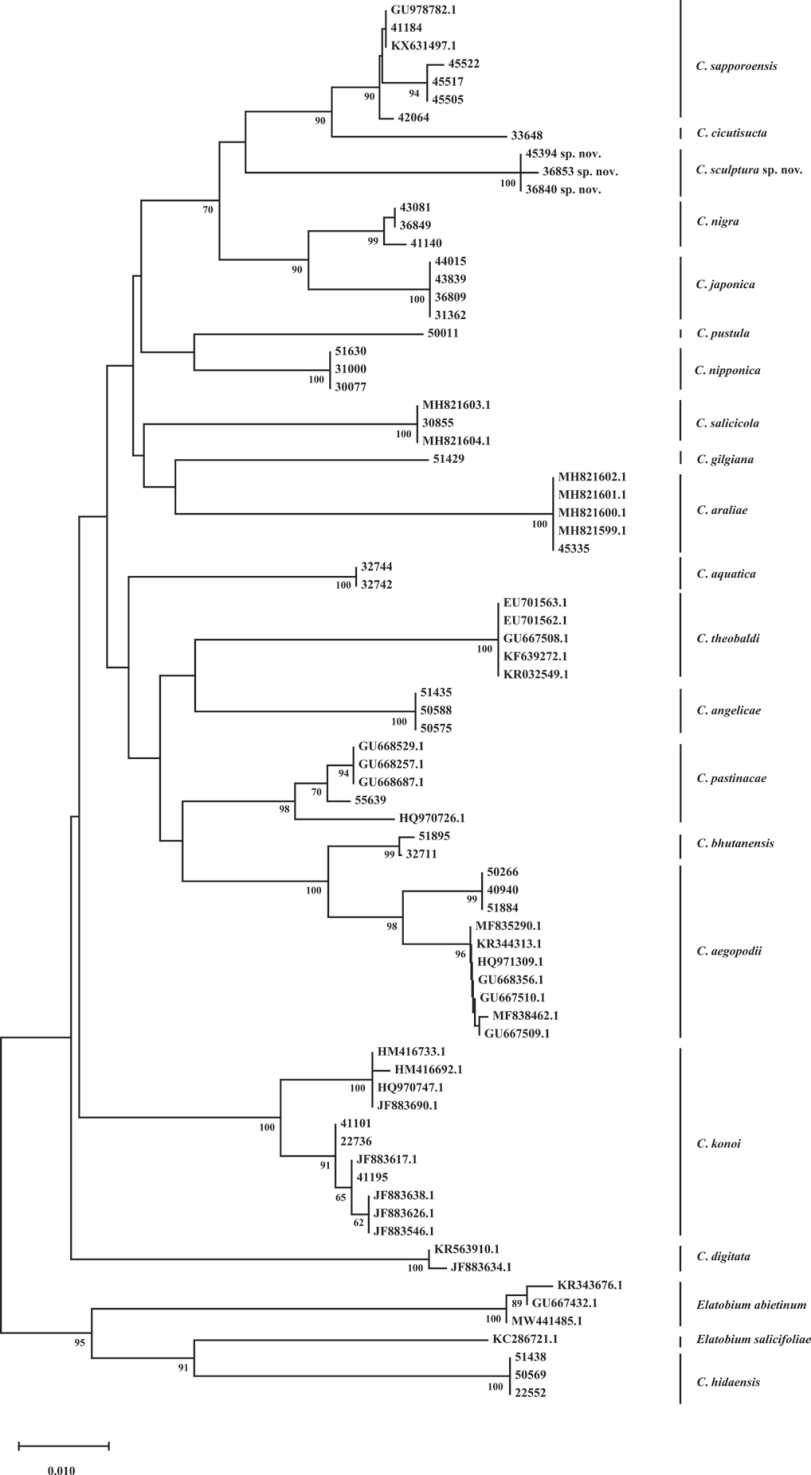
Neighbor-joining tree for *Cavariella* and *Elatobium* samples based on COI sequences.

The DNA barcodes of nineteen species of *Cavariella* were used, including the new species and others from China. The final alignments of COI sequences consisted of 658 nucleotides, including 162 parsimony-informative sites. Pairwise sequence divergences of COI among the *Cavariella* species are presented in Table 3. The interspecific genetic distances of the genus averaged 7.78% (range 2.34%–9.46%) and the divergence of the new species to others averaged 7.24% (4.84%–9.42%). The validity of each species was well-supported on NJ tree (Fig. 38). *Cavariellahidaensis* formed a clade with the genus *Elatobium*, so the NJ tree verified that the species should be removed to *Elatobium*, also, confirming morphological examination. Combination of the morphological characters and DNA barcodes supported the species of the genus *Cavariella*.

**Table 3.** Kimura’s two-parameter genetic distances of *Cavariella* species samples based on COI.

	*C.aegopodii*	*C.angelicae*	*C.aquatica*	*C.araliae*	*C.bhutanensis*	*C.cicutisucta*	*C.digitata*	*C.gilgiana*	*C.japonica*	*C.konoi*	*C.nigra*	*C.nipponica*	*C.pastinacae*	*C.pustula*	*C.salicicola*	*C.sapporoensis*	*C.sculptura* sp. nov.	*C.theobaldi*
*C.aegopodii*																		
*C.angelicae*	0.065																	
*C.aquatica*	0.066	0.055																
*C.araliae*	0.087	0.074	0.085															
*C.bhutanensis*	0.025	0.059	0.054	0.081														
*C.cicutisucta*	0.089	0.084	0.078	0.074	0.075													
*C.digitata*	0.081	0.083	0.070	0.093	0.077	0.084												
*C.gilgiana*	0.073	0.062	0.057	0.070	0.063	0.078	0.090											
*C.japonica*	0.070	0.070	0.059	0.084	0.069	0.056	0.070	0.080										
*C.konoi*	0.078	0.065	0.060	0.089	0.072	0.084	0.075	0.067	0.068									
*C.nigra*	0.074	0.065	0.059	0.069	0.070	0.057	0.070	0.072	0.023	0.068								
*C.nipponica*	0.063	0.062	0.052	0.060	0.055	0.054	0.070	0.056	0.048	0.061	0.047							
*C.pastinacae*	0.051	0.060	0.046	0.081	0.042	0.073	0.067	0.063	0.063	0.062	0.058	0.051						
*C.pustula*	0.075	0.074	0.065	0.086	0.067	0.074	0.082	0.056	0.068	0.064	0.066	0.041	0.062					
*C.salicicola*	0.077	0.066	0.059	0.076	0.067	0.072	0.084	0.062	0.062	0.068	0.062	0.054	0.068	0.066				
*C.sapporoensis*	0.076	0.078	0.058	0.074	0.064	0.027	0.064	0.066	0.043	0.071	0.047	0.050	0.062	0.064	0.060			
*C.sculptura* sp. nov.	0.091	0.080	0.068	0.082	0.087	0.054	0.086	0.082	0.056	0.081	0.048	0.056	0.077	0.080	0.072	0.054		
*C.theobaldi*	0.065	0.058	0.071	0.086	0.061	0.095	0.083	0.072	0.088	0.081	0.073	0.072	0.055	0.080	0.086	0.085	0.094	

## ﻿Discussion

After checking many specimens of *Cavariella*, the genus can be distinguished by a convex frons with undeveloped antennal tubercles, the dorsum of the body is covered with irregular sculptures, abdominal tergites VIII with a supra-caudal process and two distal setae, and the siphunculus is mostly clavate. The main characters used to distinguish the species within the genus mainly focus on the ratios of PT/Ant. Vb or Ant. VIb, URS/HT II, the ratio of the supra-caudal process/cauda, and the ratio of SIPH/cauda as well as the shapes of dorsal sculptures, the supra-caudal process, and the shape of SIPH. Because most species in the genus are green or yellow-green in life and feed on the tender tips of host plants, it is difficult to distinguish them using their ecology and habits. The DNA barcodes have successfully divided them, so it can assist to identify the species in the genus easily.

### ﻿The taxonomic status of Cavariella (Cavariellinepicauda) hidaensis Takahashi

The species was first described by Takahashi in 1961 based on the body dorsum having wavy and semicircular wrinkles; the SIPH was long, cylindrical, and not swollen, and feeding on *Salix*. However, the species has no supra-caudal process on ABD TERG VIII and with four setae on it, so there are no typical characters to place it into *Cavariella* Del Guercio. Miyazaki (1971) considered this species as belonging to *Elatobium* Mordvilko based on the head bearing prominent antennal tubercles which were higher than the median tubercle; SIPH long and cylindrical, curved outward distally; cauda long, conical, and constricted at the median; ABD TERG VIII flat with four setae and without a supra-caudal process. However, the classification status change of the species was not accepted by Blackman and Eastop (2022) or Favret (2022), and *Cavariellahidaensis* still remains in *Cavariella*. We have combined morphological characters and DNA barcoding to confirm that the species should be placed in *Elatobium* (Fig. 38). Therefore, *Cavariellahidaensis* Takahashi, 1961 is transferred to *Elatobiumhidaensis* (Takahashi, 1961).

## Supplementary Material

XML Treatment for Cavariella

XML Treatment for Cavaraiellia

XML Treatment for Cavariella (Cavaraiellia) aquatica

XML Treatment for Cavariella

XML Treatment for Cavariella aegopodii

XML Treatment for Cavariella angelicae

XML Treatment for Cavariella araliae

XML Treatment for Cavariella bhutanensis

XML Treatment for Cavariella gilgiana

XML Treatment for Cavariella japonica

XML Treatment for Cavariella konoi

XML Treatment for Cavariella lhasana

XML Treatment for Cavariella nigra

XML Treatment for Cavariella nipponica

XML Treatment for Cavariella pastinacae

XML Treatment for Cavariella pustula

XML Treatment for Cavariella salicicola

XML Treatment for Cavariella sculptura

XML Treatment for Cavariellinepicauda

XML Treatment for Cavariella (Cavariellinepicauda) cicutisucta

XML Treatment for Cavariella (Cavariellinepicauda) sapporoensis

## ﻿Additional information

### Author ORCIDs

Ying Xu https://orcid.org/0000-0002-8950-7718

Jing Chen https://orcid.org/0000-0002-7584-5249

Li-Yun Jiang https://orcid.org/0000-0002-2527-9613

Ge-Xia Qiao https://orcid.org/0000-0002-7300-6812

### Data availability

All of the data that support the findings of this study are available in the main text.
